# Associations between digital and automated work and psychosocial outcomes: Cross-sectional results from the Gutenberg Health Study

**DOI:** 10.1007/s00420-026-02252-0

**Published:** 2026-11-10

**Authors:** Alice Freiberg, Matthias Nübling, Julian Chalabi, Stephanie Drössler, Janice Hegewald, Karla Romero Starke, Hans-Joachim Lincke, Sylvia Jankowiak, Merle Riechmann-Wolf, Pavel Dietz, Manfred Beutel, Nobert Pfeiffer, Karl J. Lackner, Philipp Lurz, Emilio Gianicolo, Philipp S. Wild, Andreas Seidler

**Affiliations:** 1 Institute and Policlinic of Occupational and Social Medicine, Faculty of Medicine, Technische Universität Dresden, Fetscherstraße 74, Dresden, 01307 Germany; 2 Freiburg Research Centre for Occupational Sciences, Bertoldstraße 63, Freiburg, 79098 Germany; 3 Preventive Cardiology and Preventive Medicine, Department of Cardiology, University Medical Center of the Johannes Gutenberg University Mainz, Langenbeckstraße 1, Mainz, 55131 Germany; 4 Division Work and Health, Federal Institute for Occupational Safety and Health (BAuA), Nöldnerstraße 40–42, Berlin, 10317 Germany; 5 Institute of Occupational, Social, and Environmental Medicine, University Medical Center of the Johannes Gutenberg University Mainz, Obere Zahlbacher Str. 67, Mainz, 55131 Germany; 6 Department of Psychosomatic Medicine and Psychotherapy, University Medical Center of the Johannes Gutenberg University Mainz, Untere Zahlbacher Str. 8, Mainz, 55131 Germany; 7 Department of Ophthalmology, University Medical Center of the Johannes Gutenberg University Mainz, Langenbeckstraße 1, Mainz, 55131 Germany; 8 Institute for Clinical Chemistry and Laboratory Medicine, University Medical Center of the Johannes Gutenberg University Mainz, Langenbeckstraße 1, Mainz, 55131 Germany; 9 Department of Cardiology – Cardiology I, University Medical Center of the Johannes Gutenberg University Mainz, Langenbeckstraße 1, Mainz, 55131 Germany; 10 Institute for Medical Biostatistics, Epidemiology and Informatics, University Medical Center of the Johannes Gutenberg University Mainz, Langenbeckstraße 1, Mainz, 55131 Germany; 11 Center for Thrombosis and Hemostasis (CTH), University Medical Center of the Johannes Gutenberg University Mainz, Langenbeckstraße 1, Mainz, 55131 Germany; 12 German Center for Cardiovascular Research (DZHK), Partner Site Rhine-Main, Mainz, Germany; 13 Institute of Molecular Biology (IMB), Ackermannweg 4, Mainz, 55128 Germany

**Keywords:** Digital Work, Digital Information, Communication Tools, Automated Work, Psychosocial outcomes, Occupational Health, Workplace, Cross-Sectional Studies

## Abstract

**Purpose:**

As technology increasingly shapes workplaces, evidence associated psychosocial effects remains limited. This study explored demographic correlates of digital and automated work, and examined associations between digital and automated work and psychosocial outcomes.

**Methods:**

A cross-sectional study was conducted within the Gutenberg Health Study (Mainz/Mainz-Bingen, Germany; 2019–2022). Digital and automated work were assessed using the GAP (Gesunde Arbeit in Pionierbranchen) module. Digital work refers to the use of digital information, digital communication tools and digital networking in work processes, whereas automated work refers to the support of work from machines and robots. Psychosocial outcomes were examined using the Copenhagen Psychosocial Questionnaire (COPSOQ). Unadjusted ordinal logistic regression models estimated odds ratios and 95% confidence intervals for primary analyses. Linear regression models adjusted for age, sex, socioeconomic status, and data collection timing estimated β coefficients and 95% confidence intervals for secondary analyses.

**Results:**

Among 5,730 participants, frequent digital work was common (≈ 80%), whereas frequent automated work was rare (≈ 6%). Digital work was associated with higher job satisfaction and lower presenteeism, but also with greater intention to leave the profession and lower work engagement. Automated work was linked to higher burnout and lower job satisfaction. Technical and skill-related demands of digital and automated work were related to burnout, presenteeism, intention to leave, and reduced job satisfaction and general health. Positive experiences of digital and automated work were consistently associated with better psychosocial outcomes, including higher job satisfaction, work engagement, and general health.

**Conclusions:**

Digital work is widely integrated, whereas automated work remains limited. Both show links to psychosocial risks and resources. Legal regulations, organizational support, and individual strategies may mitigate adverse impacts. Future research should clarify mechanisms, broaden outcome assessment beyond burnout, and evaluate preventive measures amid rapid technological change.

**Supplementary Information:**

The online version contains supplementary material available at https://doi.org/10.1007/s00420-026-02252-0.

## Introduction

Technology and digitalization are among the most important drivers of the future of work, alongside other important factors such as climate change, urbanisation, and globalisation (Ghislieri et al. 2018; Schulte et al. 2020). Developments associated with the Fourth Industrial Revolution (Industry 4.0), including automation, interconnection, and computerization, are transforming workplaces across multiple sectors such as healthcare, education, manufacturing, agriculture, IT, and the public sector (Alshamsi et al. 2024; Cavicchioli et al. 2025; Gebhardt et al. 2025a, b; Håkansta et al. 2025; Hayden et al. 2022; Lindholm et al. 2024; Meyer et al. 2021; Nam and Park 2025; Scheepers et al. 2022; Schlicht et al. 2025; Schulte et al. 2020; Sınmaz et al. 2025; Tu et al. 2025; Virtanen et al. 2023; Wei and Li 2022; Wosny et al. 2023; Xie and Yang 2025; Zeike et al. 2019). Specific examples of Industry 4.0 technologies include collaborative robots in industrial manufacturing, high-performance speech recognition and text processing applications, intelligent image recognition software for medical diagnostics, and digital systems supporting the well-being of elderly people in long-term care (BMAS 2019).

In this study, the terms ‘digital work’ and ‘automated work’ are used, whereby digital work refers to the use of digital information, digital communication tools and digital networking in work processes, whereas automated work refers to the support of work from machines and robots.

Digital work encompasses a broad range of technologies and applications. These include devices, tools, and systems that are controlled or supported by computers, such as the internet of things, technical assistant systems (e.g. data glasses), wearables, or the integration of different software programs (e.g. personal requirements planning linked to time recording). Digital work also includes digital media and communication tools, such as email programmes, social networks, blogs, chats, instant messengers, paperless documentation via tablet, smartphone, notebook, PC, system display/terminals, information sharing systems, and software for telephone or video conferencing (Drössler et al. 2019).

In a German workforce sample, 42.1% of employees reported an increased workload attributable to new technologies (Pavlista et al. 2024). The proliferation of emails, chat systems, and software applications resulted in heightened information inflow, which was linked to additional demands in documentation and information management, while simultaneously creating expectations of constant availability and rapid responsiveness.

Such technology-driven changes alter work organisation and requirements for employees, impacting health in both positive and negative ways (Arana-Landín et al. 2023; Bretschneider et al. 2018; Lindholm et al. 2024; Meyer et al. 2021). Psychological outcomes are particularly relevant in this context, as aspects such as mental demands, psychological pressure, and adaptation difficulties have been identified as key occupational risk factors in Industry 4.0 (Bispo and Amaral 2024; Leso et al. 2018; Schulte et al. 2020).

Negative psychosocial effects of digital and automated work have been consistently reported in systematic reviews. Borle et al. (2021) highlighted the role of technostress, defined as negative technology-related experiences stemming from (technology-)overload, invasion, complexity, insecurity, and uncertainty (Dragano and Lunau 2020), and found associations with burnout, heightened stress levels, and negative emotions. Zolg et al. (2021) reported that software and hardware problems were associated with stress, psychosomatic complaints, lower organizational commitment, reduced career satisfaction, and higher turnover intention. Kotera and Correa Vione (2020) showed that new ways of working, where employees have autonomy over work time and location supported by media technologies, can blur work-home boundaries, increase fatigue, and heighten mental load. Bispo and Amaral (2024) highlighted that the most significant psychological consequences of working in Industry 4.0 were stress, anxiety, mental fatigue, and a sense of being replaced by technology.

While negative effects are well-documented, research also highlights potential benefits on the psychosocial level. Zolg et al. (2021) found that digital tools can improve job satisfaction, while Borle et al. (2021) reported that technostress can increase work engagement, possibly because some employees perceive technological demands as challenges or opportunities for growth rather than as threat, depending on individual factors such as technology acceptance, openness to experience, qualification level, or socioeconomic background. Kotera and Correa Vione (2020) also found that new ways of working can enhance work engagement, and also work-related flow, and connectivity among staff.

Despite growing interest, research in this area is still limited and not yet well established (Drössler et al. 2018b; Zolg et al. 2021). Studies on the psychosocial impact of working with digital and automated technologies mostly focus on burnout (see (Alshamsi et al. 2024; Bernburg et al. 2025; Cheng et al. 2021; Kaltenegger et al. 2024; Niehaus et al. 2022; Tu et al. 2025). Other relevant psychosocial effects such as job satisfaction, work engagement, or intention to leave the profession/job are scarce or missing.

Thus, this study examined how digital and automated work aspects differed according to age, sex, and socioeconomic status. It also explored associations between different aspects of digital and automated work (degree, demands, and experience) and a range of psychosocial outcomes, namely work engagement, job satisfaction, intention to leave profession/job, presenteeism, inability to relax, burnout symptoms, and general health in a population-based sample within the Gutenberg Health Study (GHS) in Germany.

## Methods

### Design

To answer the research questions, cross-sectional data from the 10- and 12.5-year follow-ups of the GHS (2017–2022) – a population-based, single-centre, prospective cohort study conducted in the Rhine-Main region of Germany (Wild et al. 2012) – were used. For the present analysis, we focused on the period 2019–2022, during which all variables relevant to our study were collected in both follow-ups. The primary aim of the GHS study was to improve the prediction of risk factors associated with cardiovascular diseases, cancers, ophthalmic diseases, metabolic diseases, immunological diseases, and mental disorders.

The study adheres to the principals of Good Clinical Practice (GCP) and Good Epidemiological Practice (Hoffmann et al. 2019) as well as the Declaration of Helsinki, and complies with the obligations of the German Federal Data Protection Act. The Ethics committee of the Rhineland-Palatinate Chamber of Physicians and the Data Protection Officer of the Johannes Gutenberg University Mainz checked the study documents, and a positive ethics vote was granted (reference number: 837.020.07(5555). Written informed consent was obtained from all participants.

### Participants

At baseline, 35,008 residents of the city of Mainz and the district of Mainz-Bingen were randomly selected from local population registration offices, from a target population of women and men aged 35 to 74 years (*n =* 210,867). The selection was stratified by sex, residence, and age. A total of 15,010 participants were enrolled at baseline between 2007 and 2012 (response: 52.2%) (Daubenbüchel 2014). Generally, individuals who were physically or mentally unable to visit the study centre, or who had insufficient German language skills, were excluded. For our specific study topic, we excluded persons who were not employed at the 10- and 12.5-year follow-ups, and who did not complete the Copenhagen Psychosocial Questionnaire (COPSOQ) questionnaire (including the GAP module) at these follow-ups (*n* = 2.263).

### GAP module

The GAP module is a questionnaire module developed by the Institute and Policlinic of Occupational and Social Medicine of the TU Dresden, Germany, as part of the project “Healthy Work in Pioneer Industries” (Gute Arbeit in Pionierbranchen = GAP) (Drössler et al. 2019). The formulation and selection of the questions (items) were based on the results of a systematic review (Bretschneider et al. 2018, 2020) as well as findings from two case studies conducted in companies in the semiconductor industry (Drössler et al. 2018a). The GAP module records demands that can occur in connection with digital and automated work. The GAP questions can be used as an additional module to conventional procedures for the risk assessment of technology-related demands at work, and in research for investigating occupational health issues. It was developed as a supplement to the COPSOQ procedure. GHS participants received an abbreviated version of the original GAP module (comprising 11 instead of 21 questions) (see Supplementary Table 1).

### Degree of digital and automated work

The degree of digital and automated work was measured with four items from the GAP module: 1) ‘use of digital information’, 2) ‘use of digital communication tools’, 3) ‘digital networking of work processes’, and 4) ‘support from machines/robots’ (Drössler et al. 2019; Magister et al. 2019). Each item could be answered using one of the following five response options: ‘always’, ‘often’, ‘sometimes’, ‘seldom’, or ‘never’, expressed as a number from 1 to 5 (never: 1; always: 5). Items 1–3 can be grouped into the composite item ‘digital work’ by adding up the values of the single items 1)–3) and dividing this sum by the number of items.

### Demands related to digital and automated work

Demands related to digital and automated work were measured using six questions from the GAP module: (1) ‘unforeseeable technical faults’, (2) ‘non-influenceable technology’, (3) ‘innovations requiring knowledge acquisition’, (4) ‘handling multiple tasks simultaneously’, (5) ‘independent problem solving’ (due to new (digital and automated) technologies, particularly in the event of incidents during automated work), and (6) ‘high volumes of information’ (Drössler et al. 2019; Magister et al. 2019). Each question could be answered using one of the following five response options: ‘always’, ‘often’, ‘sometimes’, ‘seldom’, or ‘never’, but was expressed as a number from 1 to 5 (never: 1; always: 5). The items can be grouped into ‘demands arising from technical conditions’ (items 1 and 2) and ‘demands on personal skills’ (items 3–6). For both composite items, the values of the respective individual items are summed up and divided by the number of items in the composite.

If participants reported experiencing any of these demands, they were additionally asked whether it was related to digital or automated work. The response options were ‘rather yes’, neither yes nor no’, or ‘rather no’.

### Perceived experience of digital and automated work

The perceived experience of the digital and automated work was originally measured using eight questions, but was reduced to one question for the sake of brevity (Drössler et al. 2019; Magister et al. 2019). This item could be answered using one of the following five response options: ‘negative’, ‘rather negative’, ‘neutral’, ‘rather positive’ or ‘positive’, but was expressed as a number from 1 to 5 (negative: 1, positive: 5).

### Demographic variables

Age was recorded as a continuous variable in years. Sex was coded as a dichotomous variable with the categories ‘female’ and ‘male’, with ‘male’ serving as the reference category. Socioeconomic status (SES) was treated as a continuous variable, composed of an education sum score, an occupation sum score, and a household net income score, ranging from 3 to 21, with higher scores indicating a higher SES (Hahad et al. 2024; Lampert et al. 2013a, b).

### Psychosocial effects

Psychosocial effects were measured using the following constructs from the COPSOQ questionnaire: ‘Intention to leave profession/job’, ‘Job satisfaction’, ‘Work engagement’, ‘General health’, ‘Burnout symptoms’, ‘Presenteeism’, and ‘Inability to relax’ (Burr et al. 2019; Lincke et al. 2021; Nuebling et al. 2013). These constructs are treated as continuous variables as they were assessed using a Likert scale, with response options expressed as numerical values (0–100), where higher values indicate higher levels of psychosocial strain.

### Statistical analysis

Ordinal logistic regression models were used to estimate the associations between the demographics age, sex, and SES​ and different aspects of digital and automated work (degree, demands, and experience), which were treated as ordinal categorical variables, with separate unadjusted models fitted for each demographic variable to estimate the raw associations without adjusting for potential confounders. Odds ratios (ORs) and 95% confidence intervals were calculated. P-values were Bonferroni corrected to account for multiple testing.

The associations between degree, demands, and experience of digital and automated work and psychosocial effects were estimated using separate linear regression models for the total sample and stratified for sex. Beta coefficients (β) (and 95% confidence intervals) represent the average change in the outcome scale associated with a one-unit change in the exposure variable. We adjusted for sex, age, date of measurement (to account for potential temporal effects related to the COVID-19 pandemic), and SES. No correction for multiple testing was applied due to the exploratory nature of the analyses.

All analyses were conducted using the statistic software R (version 4.4.1, Vienna, Austria).

## Results

### Sample characteristics

**Fig. 1:**
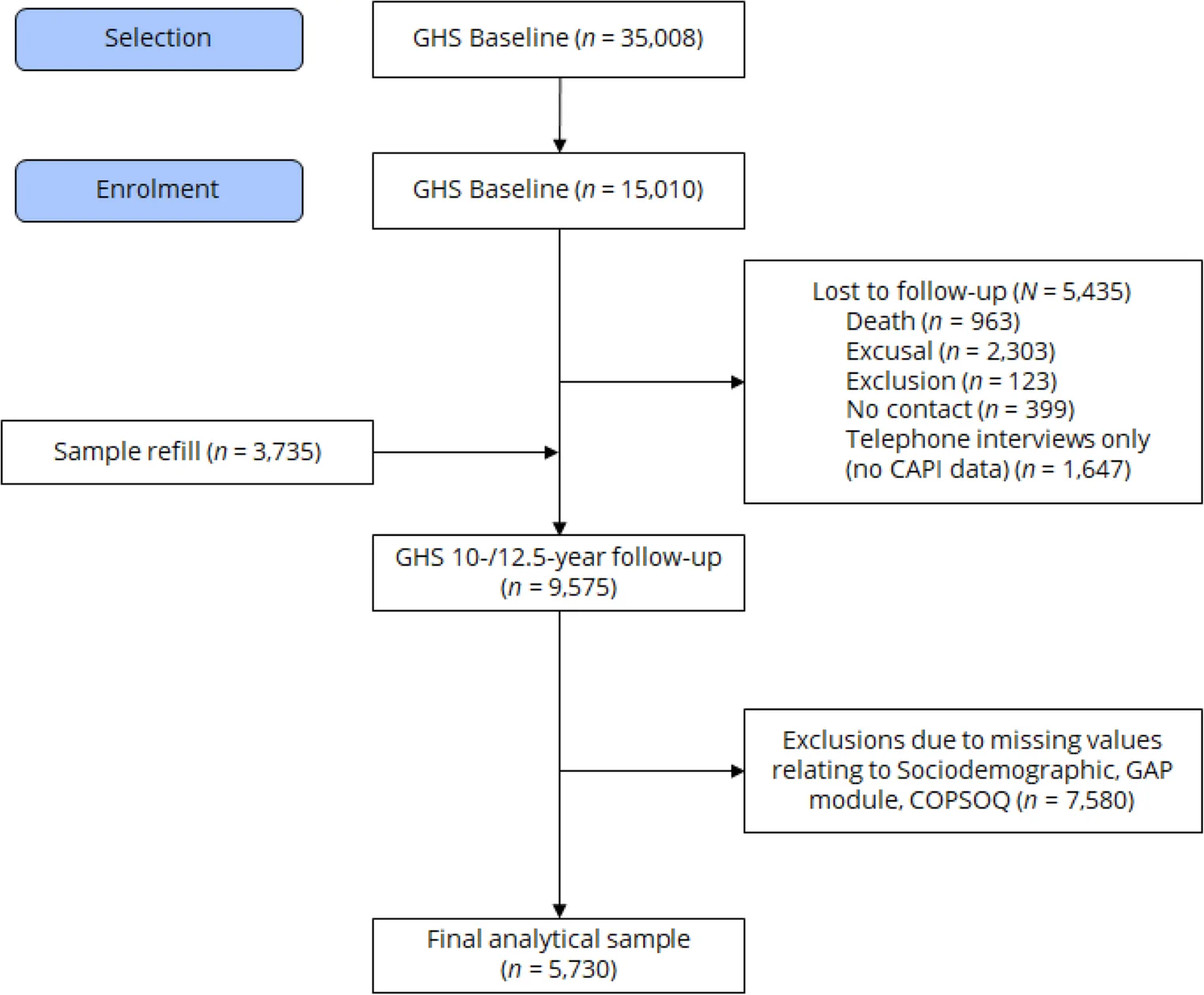
Flow diagram

5,730 participants were included in our analysis; *n* = 7,580 were excluded because essential information required for the analyses (sociodemographic variables, GAP module data, or COPSOQ items) was missing (see Fig. 1).

The study sample consisted of almost equal numbers of women (49.9%) and men (50.1%). The average age was 44.2 years (SD = 8.8). Table 1 shows further sociodemographic characteristics of the total sample regarding school and professional education, income, SES, job complexity, and occupational area. Considerable differences in sociodemographic variables were observed across job complexity levels and occupational areas (see Supplementary Tables 2 and 3).

**Table 1:** Sample characteristics. Mainz and Mainz-Bingen district. 2019–2022

Variable	*N*	%
Total	5,730	100.0
Sex		
Women	2,823	49.3
Men	2,907	50.7
Age (in years, mean (SD))	48.6 (9.9)	-
School education*		
Lower secondary school certificate	693	12.2
Intermediate secondary school certificate	1,270	22.3
Specialized higher education entrance qualification	758	13.3
General higher education entrance qualification	2,932	51.6
Other qualification	26	0.5
No qualification	6	0.1
Vocational education**		
Vocational school	1,850	32.5
Vocational college	762	13.4
University of Applied Sciences	813	14.3
University	2,003	35.2
Other qualification	106	1.9
No qualification	150	2.6
SES (categories)***		
Low	90	1.6
Intermediate	1,998	35.9
High	3,473	62.5

*Abbreviations*: *SD* standard deviation, *SES *socioeconomic status

*The number of participants (n = 5,685) is lower than the total population (n = 5,730) due to missing values

**The number of participants (n = 5,684) is lower than the total population (n = 5,730) due to missing values

***The number of participants (n = 5,561) is lower than the total population (n = 5,730) due to missing values

### Characteristics of digital and automated work

Among the overall sample, the most frequently performed digital task was using digital information, with 81.9% of respondents reporting using it often to always (see Supplementary Table 4). Similar patterns were observed for working with digital communication tools, with 79.7% often to always using such tools. Slightly lower frequencies were reported for using digital networking of work processes (73.4%). Support from automated systems or robots was only used often to always by 5.7% of respondents. The degree of digital and automated work across job complexity levels and occupational areas are shown in the Supplementary Tables 5–8.

The degree of demands relating to digital and automated work varied considerably across the six items assessed (see Supplementary Table 9). The most frequently reported demand was ‘independent problem solving’, with 64.8% of respondents indicating that they often to always encountered this. Of those who experienced this demand, more than half (50.4%) agreed that digital/automated work was the cause, while 38.4% partly agreed on this (see Supplementary Table 10). ‘Handling multiple tasks simultaneously’ often to always was reported by 59.4% of participants, with nearly half of those attributing it to digital/automated work (47.5%) and 37.4% partly agreeing. Facing ‘innovations requiring knowledge acquisition’ often to always was reported by 53.9% of participants, but of these, only 17.9% attributed this to digital/automated work, while 43.3% were ambivalent on the issue. Of the participants, 32.2% often to always dealt with ‘high volumes of information’, and nearly half of those handling this demand attributed it to digital/automated work (45.8%), while 37.6% of participants indicated that the demand was partly due to digital/automated work. Experiencing ‘non-influenceable technology’ often to always was less frequently reported (30.5%), and of those experiencing this demand, only 23.2% attributed it to digital/automated work, while 35.1% agreed on this issue to some extent. Only 11.8% of participants experienced ‘unforeseeable technical faults’ often to always, and of those, only 20.2% attributed it to digital/automated work, while 39.2% partly agreed. Demands relating to digital and automated work across job complexity levels and occupational areas are shown in the Supplementary Tables 11–16.

Overall, the majority of respondents had a positive view of working with new technologies and/or digital media (positive: 19.2% and rather positive: 43.8%), while only 4.9% gave a (rather) negative rating. A substantial proportion (32.5%) provided a neutral experience. Experience of digital and automated work across job complexity levels and occupational areas are shown in the Supplementary Table 17.

### Associations between demographics and digital and automated work

The odds of working with digital and automated technologies decreased with increasing age (see Table 2). Female sex was statistically significantly associated with less frequent use of digital and automated work, particularly regarding support from automated systems and robots at work. Higher SES levels were statistically significantly associated with more frequent use of digital information, digital communication tools, and/or digitally networked processes, but not with support from automated systems and robots.

**Table 2:** Variable specific odds ratio (OR) and 95% confidence intervals (95% CI) for different items of digital and automated work. *P*-values are correct according to Bonferroni. Mainz and Mainz-Bingen district. 2019–2022

	Use of digital information OR (95% CI)	Use of digital communication tools OR (95% CI)	Digital networking of work processes OR (95% CI)	Support from automated systems/robots OR (95% CI)
Age	0.97 (0.96 to 0.97)*p* < 0.000001*n =* 5,730	0.97 (0.96 to 0.97)*p* < 0.000001*n =* 5,720	0.97 (0.97 to 0.98)*p* < 0.000001*n =* 5,708	0.98 (0.98 to 0.99)*p* = 0.000077*n =* 5,696
Sex Women vs. men (Ref.)	0.78 (0.70 to 0.86) *p* = 0.000007 *n =* 5,730	0.79 (0.71 to 0.87) *p* = 0.000024 *n =* 5,720	0.84 (0.76 to 0.93) *p* = 0.004 *n =* 5,707	0.41 (0.35 to 0.47) *p* < 0.000001 *n =* 5,696
SES	1.21 (1.19 to 1.23) *p* < 0.000001 *n =* 5,561	1.24 (1.22 to 1.26) *p* < 0.000001 *n =* 5,551	1.18 (1.17 to 1.20) *p* < 0.000001 *n =* 5,541	1.01 (0.99 to 1.03) *p* = 1.00 *n =* 5,528

Demands related to digital and automated work showed lower odds of being reported with higher age, except for ‘non-influenceable technology’ (see Table 3). Sex differences were pronounced for the frequency of 'independent problem-solving', 'innovations requiring knowledge acquisition', 'high volumes of information', and 'unforeseeable technical faults', with women reporting such demands less frequently. No statistically significant differences were observed between the sexes for 'non-influenceable technology' and 'handling multiple tasks simultaneously'. Higher SES levels were consistently associated with more frequent technology-related demands.

**Table 3:** Variable specific odds ratio (OR) and 95% confidence intervals (95% CI) for different items of technology-related demands. P-values are correct according to Bonferroni. Mainz and Mainz-Bingen district. 2019–2022

	Unforeseeable technical faults OR (95% CI)	Non-influenceable technology OR (95% CI)	Innovations requiring knowledge acquisition OR (95% CI)	Handling multiple tasks simultaneously OR (95% CI)	Independent problem solving OR (95% CI)	High volumes of information OR (95% CI)
Age	0.98 (0.98 to 0.99)*p* < 0.000001*n =* 5,561	1.00 (0.99 to 1.00)*p* = 0.30*n =* 5,482	0.99 (0.98 to 0.99)*p* = 0.000006*n =* 5,662	0.97 (0.96 to 0.97)*p* < 0.000001*n =* 5,554	0.97 (0.97 to 0.98)*p* < 0.000001*n =* 5,576	0.98 (0.98 to 0.99)*p* < 0.000001*n =* 5,597
Sex Women vs. men (Ref.)	0.81 (0.73 to 0.89) *p* = 0.00011 *n =* 5,561	1.07 (0.97 to 1.17) *p* = 1.00 *n =* 5,482	0.65 (0.59 to 0.72) *p* < 0.000001 *n =* 5,662	0.96 (0.87 to 1.06) *p* = 1.00 *n =* 5,554	0.48 (0.44 to 0.53) *p* < 0.000001*n =* 5,576	0.67 (0.61 to 0.74) *p* < 0.000001 *n =* 5,597
SES	1.96 (1.04 to 1.07) *p* < 0.000001 *n =* 5,496	1.05 (1.03 to 1.06) *p* < 0.000001 *n =* 5,327	1.17 (1.15 to 1.19) *p* < 0.000001 *n =* 5,494	1.13 (1.11 to 1.15) *p* < 0.000001 *n =* 5,396	1.16 (1.14 to 1.17) *p* < 0.000001 *n =* 5,415	1.17 (1.15 to 1.19) *p* < 0.000001 *n =* 5,436

The odds of positively evaluating digital and automated work decreased with higher age (OR: 0.98 (95% CI: 0.98 to 0.99), p < 0.01, n = 5.571). Female participants had lower odds of evaluating work with new technologies positively compared to male participants (OR: 0.66 (95% CI: 0.60 to 0.73), p < 0.01, n = 5.571). Higher SES levels were significantly associated with more positive experiences (OR: 1.17 (95% CI: 1.16 to 1.19), p < 0.01, n = 5.408).

### Associations between digital and automated work and psychosocial outcomes

#### Degree of digital work

Working digitally, defined as using of digital information and communication tools and digitally networking of work processes, was significantly associated with a higher intention to leave the profession/job, and was also related to higher job satisfaction and lower presenteeism. No clear links were observed for work engagement, general health, burnout symptoms, or the inability to relax.

#### Degree of automated work

Support from automated systems and robots at work was associated with lower job satisfaction and work engagement and increased burnout levels. However, it showed no significant relationship with intention to leave the profession/job, general health, presenteeism, or inability to relax.

#### Unforeseeable technical faults

Unforeseeable technical faults were associated with a higher intention to leave the profession/job, more frequent burnout symptoms, more frequent presenteeism, and an increased inability to relax. They were also associated with lower job satisfaction, reduced work engagement, and poorer general health.

#### Non-influenceable technology

Exposure to non-influenceable technology at work was associated with an increased likelihood of intending to leave the profession/job and experiencing burnout symptoms, and with raised presenteeism. It was also related to lower job satisfaction, work engagement, and general health, whereas no association was found with the inability to relax.

#### Innovations requiring knowledge acquisition

Innovations requiring knowledge acquisition were associated with a higher intention to leave the profession/job, more burnout symptoms, increased presenteeism, and a greater inability to relax, and were also related to higher work engagement. Job satisfaction and general health were not significantly affected.

#### Handling multiple tasks simultaneously

Handling multiple tasks simultaneously was associated with a higher intention to leave the profession/job, and with higher burnout symptoms, greater presenteeism, and a stronger inability to relax. It was also related to lower job satisfaction and general health. No association with work engagement was shown.

#### Independent problem solving

Solving problems independently at work was associated with increased intention to leave the profession/job, burnout symptoms, presenteeism, and inability to relax. It was also related to poorer general health and reduced work engagement, whereas job satisfaction showed no significant association.

#### High volumes of information

Handling high volumes of information was associated with a higher intention to leave the profession/job, more burnout symptoms, greater presenteeism, and an increased inability to relax, along with lower job satisfaction, reduced work engagement, and poorer general health.

#### Experience of digital and automated work

Positive experience of digital and automated work was associated with lower intention to leave the profession/job, reduced burnout symptoms, decreased presenteeism, and a smaller inability to relax, and was also related to higher job satisfaction, work engagement, and general health.

#### Stratification by sex

Overall, the pattern of associations between digital and automated work and psychosocial outcomes was largely similar for women and men, with only a few differences. A higher degree of digital work tasks (i.e. the use of digital information, digital communication tools, and digital networking of work processes) was significantly associated with a higher intention to leave the profession/job and more frequent burnout symptoms in women. It was also associated with lower presenteeism in men. More frequent innovations requiring knowledge acquisition were statistically linked to a higher intention to leave the profession/job, more frequent burnout symptoms and poorer health in women only. In men, they were linked to higher job satisfaction and more work engagement. Solving problems independently more often was only significantly related to a higher intention to leave the profession/job and poorer health in women, and to more work engagement in men.

All results on the association of different aspects of digital and automated work and psychosocial outcomes for the total sample are listed in Table 4, and stratified for women and men in Supplementary tables 18 and 19.

**Table 4:** Variable specific Betas (β) and 95% confidence intervals (95% CI) for different COPSOQ items. Adjusted for sex, age, date of measurement, and SES. Mainz and Mainz-Bingen district. 2019–2022

	Intention to leave profession/job β (95% CI)	Job satisfaction β (95% CI)	Work engagement β (95% CI)	General health β (95% CI)	Burnout symptoms β (95% CI)	Presenteeism β (95% CI)	Inability to relax β (95% CI)
Use of digital information	0.81 (0.30 to 1.32) *p* = 0.0020 *n =* 5,226	0.61 (0.22 to 1.00) *p* = 0.0021 *n =* 5,098	-1.00 (-1.47 to 0.53) *p* < 0.0001 *n =* 5,304	0.10 (-0.37 to 0.39) *p* = 0.96 *n =* 5,276	0.29 (-0.21 to 0.79) *p* = 0.25 *n =* 5,279	-0.78 (-1.43 to -0.13) *p* = 0.020 *n =* 5,295	0.07 (-0.64 to 0.78) *p* = 0.84 *n =* 5,291
Use of digital communication tools	0.95 (0.46 to 1.43) *p* = 0.00015 *n =* 5,218	0.66 (0.29 to 1.03) *p* = 0.00047 *n =* 5,090	-0.97 (-1.42 to 0.53) *p* < 0.0001 *n =* 5,296	-0.04 (-0.40 to 0.32) *p* = 0.82 *n =* 5,268	0.38 (-0.10 to 0.85) *p* =0.12 *n =* 5,272	-0.56 (-1.18 to 0.06) *p* = 0.077 *n =* 5,287	0.88 (0.20 to 1.55) *p* = 0.011 *n =* 5,283
Digital networking of work processes	0.76 (0.33 to 1.19) *p* = 0.00053 *n =* 5,213	0.41 (0.09 to 0.74) *p* = 0.013 *n =* 5,086	-1.01 (-1.40 to 0.61) *p* < 0.0001 *n =* 5,292	-0.19 (-0.51 to 0.13) *p* = 0.24 *n =* 5,263	0.30 (-0.12 to 0.72) *p* =0.16 *n =* 5,266	-0.43 (-0.98 to 0.11) *p* = 0.12 *n =* 5,282	0.10 (-0.49 to 0.69) *p* = 0.75 *n =* 5,278
Degree of digital work (Composite variable)	1.02 (0.49 to 1.54) *p* = 0.00014 *n =* 5,209	0.67 (0.27 to 1.06) *p* = 0.00096 *n =* 5,082	-1.22 (-1.70 to 0.74) *p* < 0.0001 *n =* 5,288	-0.12 (-0.51 to 0.27) *p* = 0.54 *n =* 5,259	0.40 (-0.11 to 0.91) *p* = 0.13 *n =* 5,263	-0.73 (-1.39 to -0.06) *p* = 0.032 *n =* 5,278	0.40 (-0.32 to 1.12) *p* = 0.27 *n =* 5,274
Degree of automated work	0.31 (-0.29 to 0.90) *p* = 0.31 *n =* 5,195	-0.57 (-1.02 to -0.13) *p* = 0.012 *n =* 5,070	0.11 (-0.44 to 0.65) *p* =0.69 *n =* 5,273	-0.20 (-0.64 to 0.25) *p* = 0.38 *n =* 5,246	0.60 (0.02 to 1.18) *p* =0.042 *n =* 5,248	0.74 (-0.012 to 1.49) *p* = 0.055 *n =* 5,264	-0.15 (-0.97 to 0.67) *p* = 0.71 *n =* 5,260
Unforeseeable technical faults	2.73 (2.14 to 3.32) *p* < 0.0001 *n =* 5,173	-2.51 (-2.95 to -2.07) *p* < 0.0001 *n =* 5,047	-2.64 (-3.17 to -2.10) *p* < 0.0001 *n =* 5,251	-1.70 (-2.14 to -1.26) *p* < 0.0001 *n =* 5,221	3.42 (2.85 to 3.99) *p* < 0.0001 *n =* 5,226	3.47 (2.72 to 4.22) *p* < 0.0001 *n =* 5,241	2.68 (1.86 to 3.49) *p* < 0.0001 *n =* 5,237
Non-influenceable technology	1.60 (1.17 to 2.04) *p* < 0.0001 *n =* 5,042	-1.04 (-1.36 to -0.71) *p* < 0.0001 *n =* 4,930	-2.07 (-2.47 to -1.68) *p* < 0.0001 *n =* 5,114	-0.90 (-1.22 to -0.58) *p* < 0.0001 *n =* 5,089	1.86 (1.44 to 2.28) *p* < 0.0001 *n =* 5,091	0.65 (0.10 to 1.20) *p* = 0.022 *n =* 5,106	0.52 (-0.09 to 1.12) *p* = 0.092 *n =* 5,101
Demands arising from technical conditions (composite variable)	2.87 (2.27 to 3.46) *p* < 0.0001 *n =* 5,028	-2.24 (-2.69 to -1.79) *p* < 0.0001 *n =* 4,916	-3.24 (-3.79 to -2.70) *p* < 0.0001 *n =* 5,100	-1.69 (-2.13 to -1.25) *p* < 0.0001 *n =* 5,075	3.44 (2.87 to 4.02) *p* < 0.0001 *n =* 5,077	2.30 (1.54 to 3.06) *p* ​< 0.0001 *n =* 5,092	1.80 (0.97 to 2.63) *p* < 0.0001 *n =* 5,087
Innovations requiring knowledge acquisition	1.18 (0.61 to 1.75) *p* < 0.0001 *n =* 5,184	0.35 (-0.08 to 0.78) *p* = 0.11 *n =* 5,060	0.56 (0.03 to 1.08) *p* = 0.037 *n =* 5,261	-0.37 (-0.79 to 0.05) *p* = 0.086 *n =* 5,232	1.94 (1.38 to 2.49) *p* < 0.0001 *n =* 5,236	1.48 (0.75 to 2.20) *p* < 0.0001 *n =* 5,251	2.74 (1.96 to 3.53) *p* < 0.0001 *n =* 5,247
Handling multiple tasks simultaneously	2.33 (1.77 to 2.88) *p* < 0.0001 *n =* 5,109	-1.47 (-1.89 to -1.05) *p* < 0.0001 *n =* 4,987	0.22 (-0.29 to 0.72) *p* = 0.40 *n =* 5,183	-1.24 (-1.65 to -0.83) *p* < 0.0001 *n =* 5,155	3.67 (3.14 to 4.20) *p* < 0.0001 *n =* 5,157	4.35 (3.66 to 5.05) *p* < 0.0001 *n =* 5,172	5.30 (4.55 to 6.05) *p* < 0.0001 *n =* 5,168
Independent problem solving	1.45 (0.83 to 2.07) *p* < 0.0001 *n =* 5,123	-0.28 (-0.75 to 0.19) *p* = 0.24 *n =* 5,003	1.14 (0.57 to 1.70) *p* < 0.0001 *n =* 5,200	-0.68 (-1.13 to -0.22) *p* = 0.0038 *n =* 5,171	2.56 (1.96 to 3.16) *p* < 0.0001 *n =* 5,175	2.94 (2.16 to 3.73) *p* < 0.0001 *n =* 5,190	3.64 (2.79 to 4.49) *p* < 0.0001 *n =* 5,186
High volumes of information	2.71 (2.20 to 3.21) *p* < 0.0001 *n =* 5,140	-1.47 (-1.86 to -1.09) *p* < 0.0001 *n =* 5,021	-1.21 (-1.67 to -0.74) *p* < 0.0001 *n =* 5,216	-1.56 (-1.93 to -1.19) *p* < 0.0001 *n =* 5,189	4.16 (3.68 to 4.64) *p* < 0.0001 *n =* 5,191	3.41 (2.78 to 4.05) *p* < 0.0001 *n =* 5,206	4.79 (4.10 to 5.48) *p* < 0.0001 *n =* 5,202
Demands on personal skills (composite variable)	3.76 (2.99 to 4.53) *p* < 0.0001 *n =* 5,042	-1.59 (-2.17 to -1.01) *p* < 0.0001 *n =* 4,928	0.12 (-0.59 to 0.83) *p* =0.074 *n =* 5,116	-1.89 (-2.46 to -1.32) *p* < 0.0001 *n =* 5,090	4.16 (3.68 to 4.64) *p* < 0.0001 *n =* 5,092	5.85 (4.88 to 6.82) *p* < 0.0001 *n =* 5,106	7.94 (6.90 to 8.98) *p* < 0.0001 *n =* 5,102
Experience of digital and automated work	-2.12 (-2.81 to -1.42) *p* < 0.0001 *n =* 5,122	3.64 (3.12 to 4.15) *p* < 0.0001 *n =* 5,005	3.62 (2.99 to 4.25) *p* < 0.0001 *n =* 5,194	1.85 (1.33 to 2.36) *p* < 0.0001 *n =* 5,166	-3.07 (-3.74 to -2.39) *p* < 0.0001 *n =* 5,169	-4.10 (-4.98 to -3.22) *p* < 0.0001 *n =* 5,184	-1.21 (-2.17 to -0.25) *p* = 0.014 *n =* 5,180

## Discussion

### Principal findings

While most employees frequently used digital information (81,9%) and communication tools (79.7%), as well as networked processes (73.4%), frequent support from automated systems and robots remained rare (5.7%). The most common frequently occurring technology-related demands were 'independent problem-solving' (64.8%) and 'handling multiple tasks simultaneously' (59.4%). Despite these demands, digital work was experienced positively (62.0%). Younger employees, men, and those with a higher SES worked more often digitally and experienced it more favorably, but also reported higher exposure to technology-related demands.

Our findings suggest that the various aspects of digital and automated work might have different psychosocial and health-related consequences. Working digitally was associated with a higher intention to leave the profession/job, but also with higher job satisfaction and lower presenteeism. Automated work was linked to lower job satisfaction and work engagement as well as increased burnout levels.

Demands arising from technical conditions, namely unforeseeable technical faults and non-influenceable technology, was associated with an increased intention to leave the profession/job and a higher likelihood of experiencing burnout symptoms, as well as higher presenteeism. These demands were also linked to decreased job satisfaction, work engagement, and general health. Additionally, unforeseeable technical faults were related to an increased inability to relax.

All four demands on personal skills (innovations requiring knowledge acquisition, handling multiple tasks simultaneously, independent problem solving, and processing high volumes of information) were found to be associated with an increased intention to leave the profession/job, presenteeism, burnout levels, and the inability to relax. They were also associated with poorer general health. Handling multiple tasks simultaneously and processcing high volumes of information were also linked to lower job satisfaction.

Conversely, a positive experience with digital and automated work was associated with beneficial psychosocial effects, including a reduced intention to leave the profession/job, fewer burnout symptoms, lower presenteeism, a reduced inability to relax, and higher job satisfaction, work engagement, and general health.

### Digital and automated work

Regarding the mean score of technology-related demands Drössler et al. (2019), the developers of the GAP module, stated that a value above 3.5 reflects high demands and warrants caution. 'Independent problem solving' and 'handling multiple tasks simultaneously' – the two demands that occurred most frequently in the GHS sample – had mean scores above this value. At the subgroup level of this GHS sample, only complex specialist and complex activities, as well as all occupational areas except for traffic, logistics, safety and security, and agriculture, forestry, farming, and gardening (the latter only for 'handling multiple tasks simultaneously') exceeded this threshold.

Our study is only the second study to apply the GAP module to explore how new technologies and digital media are used in the workplace. The first exploratory study, conducted by Magister et al. (2019), employed a convenience sample of 74 employees from a medium-sized German company specializing in robotics and automation solutions. The use of and demands relating to new technologies at work were almost identical in both samples.

In contrast to our finding that younger employees were more likely to work with new technologies, Magister et al. (2019) found no age-related differences in experiences of working with new technologies when using the GAP module. This may be because the surveyed manufacturing company might attract employees who are generally positive about new technologies, and the changes they bring, regardless of age. However, they did not investigate any associations between other demographics and GAP items.

### Association of digital and automated work and psychosocial effects

Most studies investigating the association between technology-related work characteristics and psychosocial effects focused on burnout, whereas other psychosocial effects have been examined only sporadically. However, results regarding the impact of digital and automated work on burnout are inconclusive, with some studies reporting no significant association and others indicating increased risk. Magister et al. (2019) reported no statistically significant association between digital work and burnout symptoms, which is consistent with our findings. Similarly, Kaltenegger et al. (2024) did not find prospective associations between technostress and burnout symptoms. Importantly, they were the first to investigate the biological effects of work-related technostress in a prospective study among hospital staff. They found that technostress is linked to long-term changes in hypothalamic-pituitary-adrenal (HPA)-axis activity, as reflected in reduced hair cortisol levels, which contrasts with the typical expectation of HPA-axis activation under stress and is consistent with a pattern of hypocortisolism arising from chronic stress. Furthermore, no effects were observed on the inflammatory system. Importantly, these effects appeared independent of general work stress, indicating that digital work environments can have distinct physiological consequences beyond traditional occupational stressors. In contrast, Zhang et al. (2025) demonstrated that higher levels of enterprise digitalization were associated with higher levels of employee burnout. Similarly, Tu et al. (2025) and Bernburg et al. (2025) showed that technostress significantly increased burnout among teachers and dentists, respectively.

Research into links between working with automated machines and robots and burnout is also inconclusive. While Alshamsi et al. (2024) found no difference in work-related burnout between pharmacists using robotic versus manual dispensing systems, our study showed an increase in burnout symptoms. Cheng et al. (2021) even demonstrated a decrease in burnout among employees in jobs with a high probability of automation. However, Niehaus et al. (2022) reported that increasing instructions and control by machines were linked to higher levels of physical exhaustion and feelings of being drained (two indicators of burnout), suggesting that greater technological decision-making may worsen rather than improve working conditions. Overall, these findings indicate that the impact of technology on burnout varies depending on the type of technology and the work setting, with different effects observed for digital work versus automation or robotic systems.

Zhang et al. (2025) highlighted that employees’ role overload mediated the relationship between digitalization and burnout, while organizational support moderated the negative impact of digitalization on both burnout and role overload.

The finding that digital work increases job satisfaction was observed both in our study and in the study by Magister et al. (2019). However, technology-related demands decreased job satisfaction, as shown by our study and by Bernburg et al. (2025), who measured such demands as technostress. In contrast, Tu et al. (2025) found no association between technostress and job satisfaction. Overall, the results of Tu et al. (2025) suggest that burnout might function as a mediator between technostress and job satisfaction.

Magister et al. (2019) support our finding regarding the lack of a statistically significant association between digital work and general health. However, their finding that participants who work digitally more often report a greater inability to relax cannot be confirmed in our study. There are hardly any studies on the relationship between digital or automated work and work engagement or presenteeism.

Overall, our results suggest that digital and automated technologies have both adverse and beneficial psychosocial effects, depending on the type and experience of use. This pattern is consistent with findings by Lordan and Stringer (2022), who reported that automatable work was linked to a slight decline in mental health , and by Wei and Li (2022), who found that using artificial intelligence (AI) at work reduced depressive symptoms among manufacturing workers. 

### Implications for research and practice

As technological development in the workplace is expected to continue, it is crucial to consider how occupational safety and health (OSH) can adapt. This may involve policy and legislative updates, control measures, ergonomic improvements, and staff training (Lindholm et al. 2024). A scoping review by Rohwer et al. (2022) on the prevention of work-related technostress, found some studies on resource and coping strategies, but none on preventive measures. Their review highlights resources as an entry point for reducing technostress and underscores the role of leadership and organizational support in providing clear communication (e.g., policies or guidelines) and technical assistance (e.g., IT experts or help desks). The preventive value of organizational support was also emphasized by Zhang et al. (2025). Along these lines, Molino et al. (2021) stressed the importance of fostering positive attitudes towards new technologies by offering supervisor support and clarifying roles through communication, information, and training programs.

Older employees often find it more difficult to keep pace with emerging technologies, such as AI, which can create job insecurity and adversely affect mental health. Targeted organizational strategies, including tailored education, participatory implementation processes, and supportive, age-inclusive environments, may help address these challenges (Hossain et al. 2025; Molino et al. 2021).

Beyond organisational measures, Rohwer et al. (2022) identified personal resources such as self-efficacy, mindfulness, and a sense of control. Seeking support from colleagues appeared as a promising problem-focused coping strategy, while distress venting, 'digital detox', distancing from information technology, and positive reframing were highlighted as potentially effective emotion-focused strategies.

With regard to the psychosocial consequences of working with new technologies, some scholars also call for legal measures alongside individual and organizational action. For example, in the context of AI-based robots in the workplace, Jarota (2021) argues for an expansion of EU legislation to include clear guidelines on the use of intelligent robots, including definitions, communication procedures between employees and AI systems, and updates to OSH training for employers. Since then, the EU Artificial Intelligence Act (Regulation (EU) 2024/1689) has introduced a legal definition of AI systems (Art. 3) and obligations regarding AI literacy and training (Art. 4). However, detailed provisions on workplace communication with AI systems or OSH-specific training for employers are still lacking.

Some researchers even expect a fifth industrial revolution to build on the fourth with a more sustainable, resilient and human-centric approach. This would mean adapting technology to the needs of employees rather than the other way around (Lindholm et al. 2024). To achieve this, design-oriented principles of human factors and ergonomics should be applied in practice.

Research into the links between digital and automated work and workers’ mental wellbeing is still at an early stage (Molino et al. 2021). Although most studies focus on the antecedents and consequences of psychosocial dimensions of technology use at work, the mechanisms underlying these relationships remain insufficiently understood. Future studies should examine outcomes beyond burnout, for instance, work engagement, general health, and job satisfaction, and employ longitudinal or mixed-method designs to clarify causal pathways. Moreover, there is an urgent need for intervention studies on behavioral and structural preventive measures to mitigate the psychosocial impact of digital and automated work (Rohwer et al. 2022).

### Strengths and limitations of the study

A key strength of this study is that the GAP module was applied for the first time in a population-based sample of the German working population, which allows for population-wide inferences beyond specific occupational groups and enhances the generalizability of our findings in the national context. All data were derived from the GHS, an established, prospective cohort.

The GAP module evaluates various aspects of digital and automated work, differentiating between degree, demands, and experience, and thus allowing detailed analysis. The instruments used in this study, GAP module and COPSOQ questionnaire, have been evaluated in validation studies and demonstrated acceptable validity, reliability, and objectivity (Lincke et al. 2021; Magister et al. 2019). However, the validation of the GAP module refers to its long version; the short version used in this study has not been formally validated but was derived from the validated instrument.

In our regression analysis on the association with different aspect of digital and automated work and psychosocial effects, we adjusted for key demographics (age, sex, SES). As part of the data was collected during the COVID-19 pandemic, which may have influenced the results, we also adjusted for date of measurement to account for potential temporal effects related to the COVID-19 pandemic.

The cross-sectional design of this study precludes causal inference. While associations between digital and automated work and psychosocial effects were observed, temporality cannot be determined. As part of the 15-year follow-up of the Gutenberg Health Study, we therefore plan to investigate these associations longitudinally.

Given the rapid pace of technological change, any operationalization of digital and automated work is necessarily time-bound. Our study covered key technologies up to 2019, but did not include more recent innovations such as AI or large language models (Babashahi et al. 2024; Howard et al. 2025; Moreira et al. 2025; Naranjo et al. 2025). This should be considered when comparing our results with more recent studies.

Comparability with other studies is further challenging, as different instruments have been used to assess technology-related demands. For example, while our study employed the GAP module, other studies have relied on the technostress framework. These conceptual differences may partly account for heterogeneous findings across studies.

All measures were based on self-report, which may introduce reporting bias due to individual perceptions, memory effects, or social desirability. Nevertheless, subjective appraisal is a central element of psychosocial work factors and therefore also of relevance for the outcomes under study.

## Conclusions

Digital and automated work has become an increasingly central part of the workplace. Our study highlights the widespread integration of frequent digital work among a population-based sample of German workers, though frequent automated work remains scarce. It also suggests that working with digital and automated technologies can have negative psychosocial consequences.

However, these findings should be interpreted with caution, as the cross-sectional study design precludes causal inference and does not allow conclusions about the temporal direction of the observed associations. Furthermore, the rapid development and increasing use of artificial intelligence (AI), including large language models, may have substantially changed the nature of digital work and its associated psychosocial demands since our data were collected.

The negative psychosocial consequences of digital and automated work, such as burnout, observed in our study and also reported in research on digitalization, technostress, and newer technologies such as AI, highlight the need for further research into the underlying mechanisms and the factors contributing to these adverse effects. Such research is essential to inform the development of targeted preventive occupational safety and health (OSH) approaches at multiple levels. Potential measures include legal interventions, for example guidelines on safe use of technologies and OSH training for employers, or organizational measures, such as clear communication, targeted training programs, technical support, and supportive and inclusive leadership.

Overall, although our findings add to a robust body of research on psychosocial consequences of working with digital and automated technologies, future studies should examine the underlying mechanisms and explore outcomes beyond burnout. Research on structural and behavioral preventive strategies is particularly urgent given rapid innovations such as AI, robotics, wearables, and other emerging technologies. Addressing the psychosocial challenges of working with digital and automated technologies is essential to ensure employee well-being across the modern workplace.

## Supplementary Information

Below is the link to the electronic supplementary material.

Supplementary Material 1

## Data Availability

The analysis presents clinical data of the Gutenberg Health Study (GHS), a large-scale population-based cohort with ongoing follow-up examinations. This project constitutes a major scientific effort with high methodological standards and detailed guidelines for analysis and publication to ensure scientific analyses of highest quality. Due to the Statutes and Rules of Procedure of the GHS, data are not made available for the scientific community outside the established and controlled workflows and algorithms. To meet the general idea of verification and reproducibility of scientific findings, we offer access to data at the local database in accordance with the ethics vote upon request at any time. The GHS steering committee convenes once a month and decides on access of internal and external researchers and use of the data and biomaterials based on a research proposal. Interested researchers can make their requests to the Principal Investigators of the Gutenberg Health Study (email: ghs@unimedizin-mainz.de). More information and contact details are available on: https://www.unimedizin-mainz.de/ghs/en/informationen-for-scientists/access-to-study-data-and-biomaterial.html.
