# Supplementary material for: Associations between digital and automated work and psychosocial outcomes: Cross-sectional results from the Gutenberg Health Study
Source: Int Arch Occup Environ Health. 2026 Nov 10;99(9):63. doi: 10.1007/s00420-026-02252-0 (PMC13751699; doi:10.1007/s00420-026-02252-0)
Supplement: Supplementary file 1 [file 420_2026_2252_MOESM1_ESM.docx]

#### **Supplementary table 1: GAP module**

| **Item** | **Question** |
| --- | --- |
| **Degree of digital and automated work** | |
| **Use of digital information** (item 1) | For my work, I use information and data that are digitally available to me (e.g., product, quality, customer, and order information presented via tablet, smartphone, notebook, PC, or facility display). |
| **Use of digital communication tools** (item 2) | My work requires the use of digital communication tools (e.g., email programs, social networks, blogs, chats, instant messengers, or similar applications). |
| **Digital networking of work processes** (item 3) | My work processes are characterized by digital networking (exchange of digital information and data, e.g., via the internet, integration of various software systems). |
| **Digital work** (composite variable) | (Item 1+item 2+item 3)/3 |
| **Support from automated systems/robots** (item 4) | I work with automated systems/robots (computer-controlled machines that independently execute work processes). |
| **Demands related to digital and automated work** | |
| **Unforeseeable technical faults** (item 1) | Unforeseeable technical disruptions occur in my work. |
| **Non-influenceable technology** (item 2) | My work is determined by technology that I cannot influence. |
| **Demands arising from technical conditions** (composite variable) | (Item 1+item 2)/2 |
| **Innovations requiring knowledge acquisition** (item 3) | In my work, innovations occur that require me to acquire new knowledge. |
| **Handling multiple tasks simultaneously** (item 4) | In my work, I have to complete many tasks simultaneously. |
| **Independent problem solving** (item 5) | In my work, problems arise that I have to solve independently. |
| **High volumes of information** (item 6) | In my work, I have to deal with an overwhelming amount of information. |
| **Demands on personal skills** (composite variable) | (Item 3+item 4+item 5+item 6)/4 |
| **Experience of digital and automated work** | |
| **Experience of digital and automated work** | How would you rate your experience of working with new technologies and/or digital media? |

The items were first translated using DeepL (www.deepl.com) and subsequently refined by a native speaker.

#### **Supplementary table 2: Sample characteristics according to job complexity. Continuous variables tested with t test (or in case of skewness > 1 with U-Test. Binary variables are tested with Chi-squared test. Mainz and Mainz-Bingen district. 2019–2022.**

| **Variable** | **Unskilled activities**  (*n* = 78) | **Specialist activities**  (*n* = 1,051) | **Complex specialist activities**  (*n* = 938) | **Highly complex activities**  (*n* = 1,403) | **p for group differences** |
| --- | --- | --- | --- | --- | --- |
| **Sex** |  |  |  |  |  |
| Women | 64.1 % (50/78) | 59.0 % (620/1,051) | 48.6 % (456/938) | 43.3 % (607/1,403) | **<0.0001** |
| Men | 35.9 % (28/78) | 41.0 % (431/1,051) | 51.4 % (482/938) | 56.7 % (796/1,403) |  |
| **Age** (in years, mean (SD)) | 46.2 (9.3) | 45.2 (9.2) | 43.5 (8.5) | 43.9 (8.6) | **0.00017** |
| **School education** |  |  |  |  |  |
| Lower secondary school certificate | 32.1 % (25/78) | 18.4 % (193/1,051) | 6.1 % (57/937) | 1.1 % (16/1,403) |  |
| Intermediate secondary school certificate | 28.2 % (22/78) | 40.2 % (422/1,051) | 20.6 % (193/937) | 5.0 % (70/1,403) |  |
| Specialized higher education entrance qualification | 10.3 % (8/78) | 13.6 % (143/1,051) | 19.2 % (180/937) | 11.0 % (154/1,403) |  |
| General higher education entrance qualification | 29.5 % (23/78) | 27.0 % (284/1,051) | 53.5 % (501/937) | 82.8 % (1161/1,403) |  |
| Other qualification | 0.0 % (0/78) | 0.0 % (1/1,051) | 0.0 % (1/937) | 0.0 % (0/1,403) |  |
| No qualification | 0.0 % (0/78) | 0.0 % (8/1,051) | 0.0 % (5/937) | 0.0 % (2/1,403) |  |
| **Vocational education** |  |  |  |  |  |
| Vocational school | 57.7 % (45/78) | 63.9 % (671/1,050) | 23.6 % (221/937) | 5.1 % (72/1,403) |  |
| Vocational college | 10.3 % (8/78) | 14.7 % (154/1,050) | 20.0 % (187/937) | 4.1 % (58/1,403) |  |
| University of Applied Sciences | 2.6 % (2/78) | 6.4 % (67/1,050) | 19.6 % (184/937) | 19.0 % (266/1,403) |  |
| University | 15.4 % (12/78) | 9.6 % (101/1,050) | 31.7 % (297/937) | 68.9 % (967/1,403) |  |
| Other qualification | 1.3 % (1/78) | 1.3 % (14/1,050) | 2.9 % (27/937) | 1.6 % (22/1,403) |  |
| No qualification | 12.8 % (10/78) | 4.1 % (43/1,050) | 2.2 % (21/937) | 1.3 % (18/1,403) |  |
| **Income** (in Euros, mean (min/max)) | 3,125 (2,229/4,250) | 3,625 (2,875/4,750) | 4,750 (3,375/5,750) | 5,750 (4,250/6,750) | **<0.0001** |
| **SES** (continuous) |  |  |  |  | **<0.0001** |
| **SES** (categories) | 12.08 (3.26) | 13.22 (2.87) | 16.14 (2.87) | 18.54 (2.14) |  |
| Low | 7.7 % (6/78) | 2.2 % (23/1,039) | 0.0 % (3/933) | 0.0 % (1/1,393) |  |
| Intermediate | 69.2 % (54/78) | 65.8 % (684/1,039) | 29.3 % (273/933) | 5.7 % (79/1,393) |  |
| High | 23.1 % (18/78) | 32.0 % (332/1,039) | 70.4 % (657/933) | 94.3 % (1,313/1,393) |  |

**Abbreviation:** *min* minimum, *max* maximum, *SD* standard deviation, *SES* socioeconomic status

#### **Supplementary table 3: Sample characteristics according to occupational area. Continuous variables tested with t test (or in case of skewness > 1 with U-Test. Binary variables are tested with Chi-squared test. Mainz and Mainz-Bingen district. 2019–2022.**

| **Variable** | **Agriculture, forestry, farming, and gardening**  (*n* = 55) | **Production of raw materials and goods, and manufacturing**  (*n* = 372) | **Construction, architecture, surveying, and technical building services**  (*n* = 148) | **Natural sciences, geography, and informatics**  (*n* = 328) | **Traffic, logistics, safety and security**  (*n* = 240) | **Commercial services, trading, sales, the hotel business and tourism**  (*n* = 269) | **Business organisation, accounting, law, and administration**  (*n* = 1,012) | **Health care, the social sector, teaching and education**  (*n* = 787) | **Philology, literature, humanities, social sciences and economics, media, art, culture and design**  (*n* = 229) | **p-value for group differen-ces** |
| --- | --- | --- | --- | --- | --- | --- | --- | --- | --- | --- |
| **Sex** |  |  |  |  |  |  |  |  |  |  |
| Women | 23.6 % (13/55) | 17.5 % (65/372) | 16.9 % (25/148) | 25.3 % (83/328) | 37.9 % (91/240) | 51.4 % (152/296) | 60.1 % (608/1,012) | 72.9 % (574/787) | 53.3 % (122/229) | **<0.0001** |
| Men | 76.4 % (42/55) | 82.5 % (307/372) | 83.1 % (123/148) | 74.7 % (245/328) | 62.1 % (149/240) | 48.6 % (144/296) | 39.9 % (404/1,012) | 27.1 % (213/787) | 46.7 % (107/229) |  |
| **Age** (in years, mean (SD)) | 44.3 % (10.1) | 44.1 % (8.8) | 44.4 % (9.3) | 42.7 % (7.9) | 45.4 % (8.8) | 44.5 % (9.0) | 44.4 % (8.5) | 44.3 % (9.2) | 43.9 % (8.9) | 0.66 |
| **School education** |  |  |  |  |  |  |  |  |  |  |
| Lower secondary school certificate | 27.3 % (15/55) | 14.8 % (55/372) | 26.4 % (39/148) | 2.7 % (9/328) | 19.6 % (47/240) | 16.2 % (48/296) | 4.2 % (42/1,012) | 3.8 % (30/787) | 2.2 % (5/228) |  |
| Intermediate secondary school certificate | 29.1 % (16/55) | 23.9 % (89/372) | 17.6 % (26/148) | 10.7 % (35/328) | 24.2 % (58/240) | 25.7 % (76/296) | 24.5 % (248/1,012) | 17.2 % (135/787) | 10.5 % (24/228) |  |
| Specialized higher education entrance qualification | 10.9 % (6/55) | 24.5 % (91/372) | 16.2 % (24/148) | 15.2 % (50/328) | 13.8 % (33/240) | 10.1 % (30/296) | 14.2 % (144/1,012) | 10.7 % (84/787) | 10.1 % (23/228) |  |
| General higher education entrance qualification | 32.7 % (18/55) | 36.3 % (135/372) | 39.2 % (58/148) | 70.4 % (231/328) | 41.2 % (99/240) | 47.0 % (139/296) | 57.0 % (577/1,012) | 68.0% (535/787) | 77.2 % (176/228) |  |
| Other qualification | 0.0 % (0/55) | 0.0% (2/372) | 0.0 % (1/148) | 0.9 % (3/328) | 0.8 % (2/240) | 0.7 % (2/296) | 0.0 % (1/1012) | 0.0 % (3/787) | 0.0 % (0/228) |  |
| No qualification | 0.0 % (0/55) | 0.0 % (0/372) | 0 % (0/148) | 0 % (0/328) | 0.0 % (1/240) | 0.3 % (1/296) | 0.0 % (0/1012) | 0.0 % (0/787) | 0.0 % (0/228) |  |
| **Vocational education** |  |  |  |  |  |  |  |  |  |  |
| Vocational school | 30.9 % (17/55) | 30.6 % (114/372) | 29.7 % (44/148) | 17.7 % (58/328) | 38.8 % (93/240) | 45.4 % (134/295) | 32.3 % (327/1,012) | 23.6 % (186/787) | 15.8 % (36/228) |  |
| Vocational college | 38.2 % (21/55) | 23.1 % (86/372) | 16.9 % (25/148) | 7.3 % (24/328) | 12.9 % (31/240) | 8.8 % (26/295) | 9.6 % (97/1,012) | 11.2 % (88/787) | 3.1 % (7/228) |  |
| University of Applied Sciences | 10.9 % (6/55) | 22.3 % (83/372) | 23.0 % (34/148) | 22.9 % (75/328) | 19.6 % (47/240) | 12.2 % (36/295) | 16.7 % (169/1,012) | 4.8 % (38/787) | 13.6 % (31/228) |  |
| University | 14.5 % (8/55) | 21.2 % (79/372) | 27.7 % (41/148) | 49.1 % (161/328) | 15.0 % (36/240) | 24.7 % (73/295) | 38.4 % (389/1,012) | 56.9 % (448/787) | 62.3 % (142/228) |  |
| Other qualification | 1.8 % (1/55) | 0.5 % (2/372) | 0.7 % (1/148) | 1.2 % (4/328) | 3.3 % (8/240) | 1.7 % (5/295) | 2.1 % (21/1,012) | 2.3 % (18/787) | 1.3 % (3/228) |  |
| No qualification | 3.6 % (2/55) | 2.2 % (8/372) | 2.0 % (3/148) | 1.8 % (6/328) | 10.4 % (25/240) | 7.1 % (21/295) | 0.9 % (9/1,012) | 1.1 % (9/787) | 3.9 % (9/228) |  |
| **Income** (in Euros, mean (min/max)) | 3,625 (3,125/4,750) | 4,250 (3,375/5,750) | 4,250 (3,375/5,250) | 5,250 (4,250/6,750) | 4,250 (2,875/5,750) | 4,250 (2,979/5,750) | 4,750 (3,625/6,750) | 4,750 (3,375/5,750) | 4,750 (3,375/6,750) | **0.032** |
| **SES** (continuous) | 13.31 (2.94) | 15.32 (3.23) | 15.39 (3.43) | 17.49 (2.70) | 14.19 (3.81) | 14.68 (3.78) | 16.35 (3.31) | 16.82 (3.40) | 17.28 (3.01) | **<0.0001** |
| **SES** (categories) |  |  |  |  |  |  |  |  |  |  |
| Low | 3.7 % (2/54) | 1.9 % (7/368) | 0.7 % (1/147) | 0 % (0/328) | 2.5 % (6/237) | 3.4 % (10/294) | 0.2 % (2/1002) | 0.5 % (4/784) | 0.4 % (1/226) |  |
| Intermediate | 68.5 % (37/54) | 37.2 % (137/368) | 42.2 % (62/147) | 14.3 % (47/328) | 50.2 % (119/237) | 43.9 % (129/294) | 30.9 % (310/1,002) | 25.8 % (202/784) | 20.4 % (46/226) |  |
| High | 27.8 % (15/54) | 60.9 % (224/368) | 57.1 % (84/147) | 85.7 % (281/328) | 47.3 % (112/237) | 52.7 % (155/294) | 68.9 % (690/1,002) | 73.7 % (578/784) | 79.2 % (179/226) |  |

**Abbreviation:** *min* minimum, *max* maximum, *SD* standard deviation, *SES* socioeconomic status

#### **Supplementary table 4: Degree of digital and automated work (overall sample). Mainz and Mainz-Bingen district. 2019–2022.**

| **Variable** | **Mean (SD)**  (1–5) | **Never**  (1) | **Seldom**  (2) | **Sometimes**  (3) | **Often**  (4) | **Always**  (5) |
| --- | --- | --- | --- | --- | --- | --- |
| **Use of digital information** | 4.22 (1.16) | 6.4 % (365/5,730) | 4.4 % (252/5,730) | 7.3 % (417/5,730) | 24.4 % (1,399/5,730) | 57.5 % (3,297/5,730) |
| **Use of digital communication tools** | 4.18 (1.24) | 7.8 % (448/5,720) | 5.1 % (293/5,720) | 7.3 % (417/5,720) | 20.7 % (1,186/5,720) | 59.0 % (3,376/5,720) |
| **Digital networking of work processes** | 3.97 (1.37) | 10.9 % (623/5,708) | 7.0 % (398/5,708) | 8.7 % (498/5,708) | 21.4 % (1,223/5,708) | 52.0 % (2,966/5,708) |
| **Support from automated systems/robots** | 1.34 (0.91) | 83.8 % (4,775/5,696) | 6.9 % (393/5,696) | 3.5 % (202/5,696) | 2.5 % (141/5,696) | 3.2 % (185/5,696) |

**Abbreviations:** *NA* not applicable, *SD* standard deviation

**Supplementary table 5: Degree of digital information use according to job complexity and occupational area. Mainz and Mainz-Bingen district. 2019–2022.**

| **Variable** | **Mean (SD)**  (1–5) | **Never**  (1) | **Seldom**  (2) | **Sometimes**  (3) | **Often**  (4) | **Always**  (5) |
| --- | --- | --- | --- | --- | --- | --- |
| **Job complexity*** |  |  |  |  |  |  |
| Unskilled activities | 2.99 (1.66) | 35.9 % (28/78) | 5.1 % (4/78) | 9.0 % (7/78) | 24.4 % (19/78) | 25.6 % (20/78) |
| Specialist activities | 3.97 (1.31) | 9.6 % (101/1,051) | 6.5 % (68/1,051) | 10.8 % (113/1,051) | 23.5 % (247/1,051) | 49.7 % (522/1,051) |
| Complex specialist activities | 4.42 (1.00) | 3.2 % (30/938) | 4.3 % (40/938) | 6.2 % (58/938) | 20.5 % (192/938) | 65.9 % (618/938) |
| Highly complex activities | 4.60 (0.74) | 1.1 % (15/1,403) | 1.7 % (24/1,403) | 3.7 % (52/1,403) | 22.8 % (320/1,403) | 70.7 % (992/1,403) |
| **Occupational area**** |  |  |  |  |  |  |
| Agriculture, forestry, farming, and gardening | 3.18 (1.33) | 16.4 % (9/55) | 16.4 % (9/55) | 14.5 % (8/55) | 38.2 % (21/55) | 14.5 % (8/55) |
| Production of raw materials and goods, and manufacturing | 4.28 (1.13) | 4.8 % (18/372) | 6.2 % (23/372) | 6.5 % (24/372) | 21.5 % (80/372) | 61.0 % (227/372) |
| Construction, architecture, surveying, and technical building services | 3.93 (1.27) | 9.5 % (14/148) | 5.4 % (8/148) | 11.5 % (17/148) | 30.4 % (45/148) | 43.2 % (64/148) |
| Natural sciences, geography, and informatics | 4.84 (0.45) | 0.3 % (1/328) | 0.3 % (1/328) | 0.6 % (2/328) | 12.2 % (40/328) | 86.6 % (284/328) |
| Traffic, logistics, safety and security | 3.93 (1.38) | 13.3 % (32/240) | 3.8 % (9/240) | 7.5 % (18/240) | 27.5 % (66/240) | 47.9 % (115/240) |
| Commercial services, trading, sales, the hotel business and tourism | 4.10 (1.31) | 9.8 % (29/296) | 4.7 % (14/296) | 7.8 % (23/296) | 20.6 % (61/296) | 57.1 % (169/296) |
| Business organisation, accounting, law, and administration | 4.71 (0.62) | 0.6 % (6/1,012) | 0.9 % (9/1,012) | 2.6 % (26/1,012) | 18.7 % (189/1,012) | 77.3 % (782/1,012) |
| Health care, the social sector, teaching and education | 3.89 (1.25) | 8.0 % (63/787) | 7.5 % (59/787) | 13.7 % (108/787) | 29.2 % (230/787) | 41.6 % (327/787) |
| Philology, literature, humanities, social sciences and economics, media, art, culture and design | 4.70 (0.63) | 0.4 % (1/229) | 1.7 % (4/229) | 1.7 % (4/229) | 19.2 % (44/229) | 76.9 % (176/229) |

* The mean values between job complexities differed statistically significantly according to the t-test (p-value < 0.0001).

** The mean values between occupational areas did not differ statistically significantly according to the t-test (p-value = 0.14).

**Supplementary table 6: Degree of digital communication tool use according to job complexity and occupational area. Mainz and Mainz-Bingen district. 2019–2022.**

| **Variable** | **Mean (SD)**  (1–5) | **Never**  (1) | **Seldom**  (2) | **Sometimes**  (3) | **Often**  (4) | **Always**  (5) |
| --- | --- | --- | --- | --- | --- | --- |
| **Job complexity*** |  |  |  |  |  |  |
| Unskilled activities | 2.74 (1.62) | 37.2 % (29/78) | 12.8 % (10/78) | 11.5 % (9/78) | 15.4 % (12/78) | 23.1 % (18/78) |
| Specialist activities | 3.86 (1.40) | 11.2 % (118/1,049) | 9.3 % (98/1,049) | 10.2 % (107/1,049) | 20.9 % (219/1,049) | 48.3 % (507/1,049) |
| Complex specialist activities | 4.41 (1.05) | 3.9 % (37/938) | 4.7 % (44/938) | 5.2 % (49/938) | 18.4 % (173/938) | 67.7 % (635/938) |
| Highly complex activities | 4.65 (0.71) | 1.0 % (14/1,402) | 1.4 % (20/1,402) | 3.9 % (54/1,402) | 19.3 % (270/1,402) | 74.5 % (1,044/1,402) |
| **Occupational area**** |  |  |  |  |  |  |
| Agriculture, forestry, farming, and gardening | 3.20 (1.35) | 16.4 % (9/55) | 16.4 % (9/55) | 16.4 % (9/55) | 32.7 % (18/55) | 18.2 % (10/55) |
| Production of raw materials and goods, and manufacturing | 4.20 (1.24) | 7.0 % (26/372) | 7.5 % (28/372) | 4.0 % (15/372) | 21.2 % (79/372) | 60.2 % (224/372) |
| Construction, architecture, surveying, and technical building services | 3.99 (1.32) | 10.1 % (15/148) | 5.4 % (8/148) | 10.1 % (15/148) | 24.3 % (36/148) | 50.0 % (74/148) |
| Natural sciences, geography, and informatics | 4.77 (0.66) | 1.5 % (5/328) | 0.6 % (2/328) | 1.8 % (6/328) | 11.6 % (38/328) | 84.5 % (277/328) |
| Traffic, logistics, safety and security | 3.79 (1.43) | 13.3 % (32/240) | 9.2 % (22/240) | 7.9 % (19/240) | 24.6 % (59/240) | 45.0 % (108/240) |
| Commercial services, trading, sales, the hotel business and tourism | 4.08 (1.39) | 11.9 % (35/294) | 4.1 % (12/294) | 8.8 % (26/294) | 14.6 % (43/294) | 60.5 % (178/294) |
| Business organisation, accounting, law, and administration | 4.76 (0.59) | 0.6 % (6/1,012) | 0.9 % (9/1,012) | 2.1 % (21/1,012) | 14.9 % (151/1,012) | 81.5 % (825/1,012) |
| Health care, the social sector, teaching and education | 3.82 (1.31) | 8.7 % (68/786) | 10.2 % (80/786) | 13.4 % (105/786) | 26.1 % (205/786) | 41.7 % (328/786) |
| Philology, literature, humanities, social sciences and economics, media, art, culture and design | 4.72 (0.62) | 0.9 % (2/229) | 0.9 % (2/229) | 1.3 % (3/229) | 18.8 % (43/229) | 78.2 % (179/229) |

* The mean values between job complexities differed statistically significantly according to the t-test (p-value < 0.0001).

** The mean values between occupational areas did not differ statistically significantly according to the t-test (p-value = 0.31).

**Supplementary table 7: Degree of using digital networking of work processes according to job complexity and occupational area. Mainz and Mainz-Bingen district. 2019–2022.**

| **Variable** | **Mean (SD)**  (1–5) | **Never**  (1) | **Seldom**  (2) | **Sometimes**  (3) | **Often**  (4) | **Always**  (5) |
| --- | --- | --- | --- | --- | --- | --- |
| **Job complexity*** |  |  |  |  |  |  |
| Unskilled activities | 2.45 (1.61) | 47.4 % (37/78) | 11.5 % (9/78) | 9.0 % (7/78) | 12.8 % (10/78) | 19.2 % (15/78) |
| Specialist activities | 3.68 (1.50) | 15. 6 % (163/1,047) | 10.0 % (105/1,047) | 10.2 % (107/1,047) | 18.8 % (197/1,047) | 45.4 % (475/1,047) |
| Complex specialist activities | 4.17 (1.25) | 7.5 % (70/937) | 5.9 % (55/937) | 8.5 % (80/937) | 18.8 % (176/937) | 59.3 % (556/937) |
| Highly complex activities | 4.39 (1.00) | 3.1 % (43/1,402) | 3.9 % (55/1,402) | 7.9 % (111/1,402) | 21.4 % (300/1,402) | 63.7 % (893/1,402) |
| **Occupational area**** |  |  |  |  |  |  |
| Agriculture, forestry, farming, and gardening | 2.35 (1.25) | 36.4 % (20/55) | 18.2 % (10/55) | 25.5 % (14/55) | 14.5 % (8/55) | 5.5 % (3/55) |
| Production of raw materials and goods, and manufacturing | 4.03 (1.29) | 8.1 % (30/372) | 7.8 % (29/372) | 9.4 % (35/372) | 22.0 % (82/372) | 52.7 % (196/372) |
| Construction, architecture, surveying, and technical building services | 3.61 (1.45) | 14.9 % (22/148) | 9.5 % (14/148) | 14.2 % (21/148) | 22.2 % (33/148) | 39.2 % (58/148) |
| Natural sciences, geography, and informatics | 4.73 (0.70) | 1.5 % (5/328) | 1.2 % (4/328) | 1.8 % (6/328) | 13.7 % (45/328) | 81.7 % (268/328) |
| Traffic, logistics, safety and security | 3.71 (1.50) | 16.3 % (39/240) | 8.3 % (20/240) | 9.2 % (22/240) | 20.8 % (50/240) | 45.4 % (109/240) |
| Commercial services, trading, sales, the hotel business and tourism | 3.82 (1.51) | 16.3 % (48/295) | 6.4 % (19/295) | 7.1 % (21/295) | 19.0 % (56/295) | 51.2 % (151/295) |
| Business organisation, accounting, law, and administration | 4.61 (0.76) | 1.2 % (12/1,011) | 1.7 % (17/1,011) | 5.0 % (51/1,011) | 19.1 % (193/1,011) | 73.0 % (738/1,011) |
| Health care, the social sector, teaching and education | 3.42 (1.47) | 16.6 % (130/784) | 13.4 % (105/784) | 15.3 % (120/784) | 21.3 % (167/784) | 33.4 % (262/784) |
| Philology, literature, humanities, social sciences and economics, media, art, culture and design | 4.46 (0.95) | 3.1 % (7/228) | 2.6 % (6/228) | 6.1 % (14/228) | 21.1 % (48/228) | 67.1 % (153/228) |

* The mean values between job complexities differed statistically significantly according to the t-test (p-value < 0.0001).

** The mean values between occupational areas differed statistically significantly according to the t-test (p-value = 0.011).

**Supplementary table 8: Degree of support from automated systems/robots according to job complexity and occupational area. Mainz and Mainz-Bingen district. 2019–2022.**

| **Variable** | **Mean (SD)**  (1–5) | **Never**  (1) | **Seldom**  (2) | **Sometimes**  (3) | **Often**  (4) | **Always**  (5) |
| --- | --- | --- | --- | --- | --- | --- |
| **Job complexity*** |  |  |  |  |  |  |
| Unskilled activities | 1.38 (0.97) | 85.7 % (66/77) | 1.3 % (1/77) | 3.9 % (3/77) | 7.8 % (6/77) | 1.3 % (1/77) |
| Specialist activities | 1.44 (1.08) | 82.8 % (864/1,043) | 4.8 % (50/1,043) | 3.1 % (32/1,043) | 4.1 % (43/1,043) | 5.2 % (54/1,043) |
| Complex specialist activities | 1.33 (0.84) | 82.3 % (769/934) | 8.9 % (83/934) | 4.3 % (40/934) | 2.2 % (21/934) | 2.2 % (21/934) |
| Highly complex activities | 1.33 (0.89) | 83.2 % (1,165/1,400) | 8.4 % (117/1,400) | 3.6 % (51/1,400) | 1.4 % (19/1,400) | 3.4 % (48/1,400) |
| **Occupational area**** |  |  |  |  |  |  |
| Agriculture, forestry, farming, and gardening | 1.36 (0.72) | 76.4 % (42/55) | 12.7 % (7/55) | 9.1 % (5/55) | 1.8 % (1/55) | 0.0 % (0/55) |
| Production of raw materials and goods, and manufacturing | 1.87 (1.37) | 63.9 % (237/371) | 12.9 % (48/371) | 5.9 % (22/371) | 7.0 % (26/371) | 10.2 % (38/371) |
| Construction, architecture, surveying, and technical building services | 1.22 (0.62) | 87.0 % (127/146) | 6.2 % (9/146) | 4.8 % (7/146) | 2.1 % (3/146) | 0.0 % (0/146) |
| Natural sciences, geography, and informatics | 1.71 (1.22) | 66.5 % (218/328) | 14.9 % (49/328) | 6.4 % (21/328) | 5.2 % (17/328) | 7.0 % (23/328) |
| Traffic, logistics, safety and security | 1.34 (0.91) | 84.5 % (201/238) | 6.3 % (15/238) | 2.5 % (6/238) | 4.2 % (10/238) | 2.5 % (6/238) |
| Commercial services, trading, sales, the hotel business and tourism | 1.19 (0.70) | 91.2 % (268/294) | 2.7 % (8/294) | 3.4 % (10/294) | 1.0 % (3/294) | 1.7 % (5/294) |
| Business organisation, accounting, law, and administration | 1.20 (0.67) | 88.8 % (894/1,007) | 6.1 % (61/1,007) | 2.6 % (26/1,007) | 1.2 % (12/1,007) | 1.4 % (14/1,007) |
| Health care, the social sector, teaching and education | 1.33 (0.96) | 87.0 % (682/784) | 4.3 % (34/784) | 2.3 % (18/784) | 1.8 % (14/784) | 4.6 % (36/784) |
| Philology, literature, humanities, social sciences and economics, media, art, culture and design | 1.26 (0.69) | 84.2 % (192/228) | 8.8 % (20/228) | 4.8 % (11/228) | 1.3 % (3/228) | 0.9 % (2/228) |

* The mean values between job complexities did not differ statistically significantly according to the t-test (p-value = 0.69).

** The mean values between occupational areas differed statistically significantly according to the t-test (p-value < 0.0001).

**Supplementary table 9: Degree of demands relating to digital and automated work (overall sample). Mainz and Mainz-Bingen district. 2019–2022.**

| **Variable** | **Mean (SD)**  (1–5) | **Never**  (1) | **Seldom**  (2) | **Sometimes**  (3) | **Often**  (4) | **Always**  (5) |
| --- | --- | --- | --- | --- | --- | --- |
| **Unforeseeable technical faults** | 2.48 (0.93) | 16.7 % (943/5,661) | 31.5 % (1,783/5,661) | 40.1 % (2,268/5,661) | 10.8 % (612/5,661) | 1.0 % (55/5,661) |
| **Non-influenceable technology** | 2.74 (1.27) | 21.8 % (1,193/5,482) | 22.0 % (1,204/5,482) | 25.7 % (1,409/5,482) | 21.3 % (1,170/5,482) | 9.2 % (506/5,482) |
| **Innovations requiring knowledge acquisition** | 3.46 (1.01) | 5.7 % (324/5,662) | 9.1 % (516/5,662) | 31.3 % (1,772/5,662) | 41.5 % (2,349/5,662) | 12.4 % (701/5,662) |
| **Handling multiple tasks simultaneously** | 3.56 (1.04) | 5.1 % (284/5,554) | 9.8 % (542/5,554) | 25.7 % (1,430/5,554) | 43.1 % (2,392/5,554) | 16.3 % (906/5,554) |
| **Independent problem solving** | 3.70 (0.95) | 3.4 % (192/5,576) | 6.4 % (357/5,576) | 25.3 % (1,411/5,576) | 46.5 % (2,594/5,576) | 18.3 % (1,022/5,576) |
| **High volumes of information** | 2.93 (1.13) | 11.9 % (664/5,597) | 24.3 % (1,362/5,597) | 31.3 % (1,750/5,597) | 24.2 % (1,355/5,597) | 8.0 % (466/5,597) |

**Abbreviations:** *NA* not applicable, *SD* standard deviation

**Supplementary table 10: Attribution of demands relating digital and automated work (overall sample). Mainz and Mainz-Bingen district. 2019–2022.**

| **Variable** | **Mean (SD)**  (1–3) | **Rather yes**  (1) | **Neither yes nor no**  (2) | **Rather no**  (3) |
| --- | --- | --- | --- | --- |
| **Unforeseeable technical faults** | 2.20 (0.75) | 20.2 % (1,000/4,950) | 39.2 % (1,941/4,950) | 40.6 % (2,009/4,950) |
| **Non-influenceable technology** | 2.18 (0.78) | 23.2 % (1,065/4,583) | 35.1 % (1,611/4,583) | 41.6 % (1,907/4,583) |
| **Innovations requiring knowledge acquisition** | 2.21 (0.72) | 17.9 % (948/5,310) | 43.3 % (2,298/5,310) | 38.9 % (2,064/5,310) |
| **Handling multiple tasks simultaneously** | 1.68 (0.72) | 47.5 % (2,469/5,200) | 37.4 % (1,944/5,200) | 15.1 % (787/5,200) |
| **Independent problem solving** | 1.61 (0.68) | 50.4 % (2,675/5,305) | 38.4 % (2,037/5,305) | 11.2 % (593/5,305) |
| **High volumes of information** | 1.71 (0.73) | 45.8 % (2,320/5,065) | 37.6 % (1,904/5,065) | 16.6 % (841/5,065) |

**Supplementary table 11: Degree of unforeseeable technical faults according to job complexity and occupational area. Mainz and Mainz-Bingen district. 2019–2022.**

| **Variable** | **Mean (SD)**  (1–5) | **Never**  (1) | **Seldom**  (2) | **Sometimes**  (3) | **Often**  (4) | **Always**  (5) |
| --- | --- | --- | --- | --- | --- | --- |
| **Job complexity*** |  |  |  |  |  |  |
| Unskilled activities | 2.16 (1.05) | 36.4 % (28/77) | 22.1 % (17/77) | 32.5 % (25/77) | 7.8 % (6/77) | 1.3 % (1/77) |
| Specialist activities | 2.46 (0.98) | 19.4 % (201/1,035) | 30.5 % (316/1,035) | 35.6 % (368/1,035) | 13.4 % (139/1,035) | 1.1 % (11/1,035) |
| Complex specialist activities | 2.55 (0.94) | 15.5 % (144/930) | 28.8 % (268/930) | 42.0 % (391/930) | 12.3 % (114/930) | 1.4 % (13/930) |
| Highly complex activities | 2.57 (0.86) | 11.1 % (156/1,401) | 33.3 % (466/1,401) | 43.4 % (608/1,401) | 11.6 % (162/1,401) | 0.6 % (9/1,401) |
| **Occupational area**** |  |  |  |  |  |  |
| Agriculture, forestry, farming, and gardening | 2.19 (0.87) | 22.6 % (12/53) | 41.5 % (22/53) | 32.1 % (17/53) | 1.9 % (1/53) | 1.9 % (1/53) |
| Production of raw materials and goods, and manufacturing | 2.68 (1.04) | 13.8 % (51/369) | 30.1 % (111/369) | 33.6 % (124/369) | 19.2 % (71/369) | 3.3 % (12/369) |
| Construction, architecture, surveying, and technical building services | 2.34 (0.94) | 19.7 % (29/147) | 38.1 % (56/147) | 32.0 % (47/147) | 8.8 % (13/147) | 1.4 % (2/147) |
| Natural sciences, geography, and informatics | 2.81 (0.90) | 6.4 % (21/327) | 30.0 % (98/327) | 42.5 % (139/327) | 18.3 % (60/327) | 2.8 % (9/327) |
| Traffic, logistics, safety and security | 2.54 (0.89) | 14.3 % (34/238) | 30.3 % (72/238) | 43.3 % (103/238) | 11.8 % (28/238) | 0.4 % (1/238) |
| Commercial services, trading, sales, the hotel business and tourism | 2.33 (0.91) | 21.6 % (63/292) | 32.2 % (94/292) | 37.7 % (110/292) | 8.6 % (25/292) | 0.0 % (0/292) |
| Business organisation, accounting, law, and administration | 2.50 (0.84) | 12.6 % (127/1,008) | 34.8 % (351/1,008) | 42.8 % (431/1,008) | 9.5 % (96/1,008) | 0.3 % (3/1,008) |
| Health care, the social sector, teaching and education | 2.50 (0.99) | 20.7 % (161/778) | 23.7 % (184/778) | 41.0 % (319/778) | 13.9 % (108/778) | 0.8 % (6/778) |
| Philology, literature, humanities, social sciences and economics, media, art, culture and design | 2.47 (0.82) | 13.6 % (31/228) | 33.8 % (77/228) | 44.7 % (102/228) | 7.9 % (18/228) | 0.0 % (0/228) |

* The mean values between job complexities differed statistically significantly according to the t-test (p-value = 0.00072).

** The mean values between occupational areas did not differ statistically significantly according to the t-test (p-value = 0.086).

**Supplementary table 12: Degree of non-influenceable technology according to job complexity and occupational area. Mainz and Mainz-Bingen district. 2019–2022.**

| **Variable** | **Mean (SD)**  (1–5) | **Never**  (1) | **Seldom**  (2) | **Sometimes**  (3) | **Often**  (4) | **Always**  (5) |
| --- | --- | --- | --- | --- | --- | --- |
| **Job complexity*** |  |  |  |  |  |  |
| Unskilled activities | 2.26 (1.34) | 41.6 % (32/77) | 20.8 % (16/77) | 16.9 % (13/77) | 11.7 % (9/77) | 9.1 % (7/77) |
| Specialist activities | 2.81 (1.39) | 25.1 % (253/1,008) | 18.6 % (187/1,008) | 20.3 % (205/1,008) | 22.0 % (222/1,008) | 14.0 % (141/1,008) |
| Complex specialist activities | 2.80 (1.22) | 18.7 % (173/924) | 21.8 % (201/924) | 29.0 % (268/924) | 21.9 % (202/924) | 8.7 % (80/924) |
| Highly complex activities | 2.72 (1.16) | 17.1 % (237/1,388) | 27.2 % (378/1,388) | 28.3 % (393/1,388) | 21.2 % (294/1,388) | 6.2 % (86/1,388) |
| **Occupational area**** |  |  |  |  |  |  |
| Agriculture, forestry, farming, and gardening | 1.87 (0.99) | 45.3 % (24/53) | 32.1 % (17/53) | 15.1 % (8/53) | 5.7 % (3/53) | 1.9 % (1/53) |
| Production of raw materials and goods, and manufacturing | 2.76 (1.13) | 16.3 % (60/369) | 25.2 % (93/369) | 29.5 % (109/369) | 24.1 % (89/369) | 4.9 % (18/369) |
| Construction, architecture, surveying, and technical building services | 2.25 (1.10) | 29.5 % (43/146) | 34.9 % (51/146) | 19.2 % (28/146) | 13.7 % (20/146) | 2.7 % (4/146) |
| Natural sciences, geography, and informatics | 2.91 (1.10) | 10.3 % (33/321) | 25.9 % (83/321) | 35.2 % (113/321) | 20.2 % (65/321) | 8.4 % (27/321) |
| Traffic, logistics, safety and security | 2.85 (1.35) | 21.8 % (51/234) | 20.9 % (49/234) | 20.9 % (49/234) | 23.1 % (54/234) | 13.2 % (31/234) |
| Commercial services, trading, sales, the hotel business and tourism | 2.70 (1.27) | 24.2 % (69/285) | 20.0 % (57/285) | 24.9 % (71/285) | 23.2 % (66/285) | 7.7 % (22/285) |
| Business organisation, accounting, law, and administration | 3.13 (1.26) | 13.3 % (132/995) | 18.9 % (188/995) | 24.1 % (240/995) | 28.6 % (285/995) | 15.1 % (150/995) |
| Health care, the social sector, teaching and education | 2.31 (1.19) | 33.1 % (254/767) | 24.1 % (185/767) | 25.7 % (197/767) | 12.4 % (95/767) | 4.7 % (36/767) |
| Philology, literature, humanities, social sciences and economics, media, art, culture and design | 2.92 (1.19) | 12.5 % (28/224) | 26.3 % (59/224) | 28.1 % (63/224) | 22.3 % (50/224) | 10.7 % (24/224) |

* The mean values between job complexities did not differ statistically significantly according to the t-test (p-value = 0.59).

** The mean values between occupational areas did not differ statistically significantly according to the t-test t (p-value = 0.088).

**Supplementary table 13: Degree of** **innovations requiring knowledge acquisition according to job complexity and occupational area. Mainz and Mainz-Bingen district. 2019–2022.**

| **Variable** | **Mean (SD)**  (1–5) | **Never**  (1) | **Seldom**  (2) | **Sometimes**  (3) | **Often**  (4) | **Always**  (5) |
| --- | --- | --- | --- | --- | --- | --- |
| **Job complexity*** |  |  |  |  |  |  |
| Unskilled activities | 2.58 (1.21) | 26.3 % (20/76) | 17.1 % (13/76) | 35.5 % (27/76) | 14.5 % (11/76) | 6.6 % (5/76) |
| Specialist activities | 3.16 (1.08) | 9.7% (100/1,036) | 13.5 % (140/1,036) | 36.4 % (377/1,036) | 31.9 % (330/1,036) | 8.6 % (89/1,036) |
| Complex specialist activities | 3.50 (0.95) | 4.2 % (39/930) | 9.2 % (86/930) | 29.8 % (277/930) | 46.0 % (428/930) | 10.8 % (100/930) |
| Highly complex activities | 3.79 (0.87) | 1.5 % (21/1,400) | 5.2 % (73/1,400) | 25.9 % (363/1,400) | 47.9 % (671/1,400) | 19.4 % (272/1,400) |
| **Occupational area**** |  |  |  |  |  |  |
| Agriculture, forestry, farming, and gardening | 3.00 (0.99) | 7.5 % (4/53) | 20.8 % (11/53) | 41.5 % (22/53) | 24.5 % (13/53) | 5.7 % (3/53) |
| Production of raw materials and goods, and manufacturing | 3.59 (0.92) | 3.0 % (11/368) | 8.4 % (31/368) | 27.7 % (102/368) | 48.1 % (177/368) | 12.8 % (47/368) |
| Construction, architecture, surveying, and technical building services | 3.23 (1.03) | 8.2 % (12/146) | 11.0 % (16/146) | 39.0 % (57/146) | 33.6 % (49/146) | 8.2 % (12/146) |
| Natural sciences, geography, and informatics | 4.07 (0.82) | 0.6 % (2/328) | 3.7 % (12/328) | 15.2 % (50/328) | 48.8 % (160/328) | 31.7 % (104/328) |
| Traffic, logistics, safety and security | 3.23 (1.07) | 8.9 % (21/237) | 11.8 % (28/237) | 36.3 % (86/237) | 33.3 % (79/237) | 9.7 % (23/237) |
| Commercial services, trading, sales, the hotel business and tourism | 3.18 (1.07) | 9.2 % (27/292) | 14.4 % (42/292) | 32.9 % (96/292) | 36.0 % (105/292) | 7.5 % (22/292) |
| Business organisation, accounting, law, and administration | 3.59 (0.91) | 2.9 % (29/1,010) | 7.3 % (74/1,010) | 31.3 % (316/1,010) | 45.2 % (457/1,010) | 13.3 % (134/1,010) |
| Health care, the social sector, teaching and education | 3.33 (1.11) | 8.7 % (68/778) | 11.6 % (90/778) | 30.3 % (236/778) | 36.9 % (287/778) | 12.5 % (97/778) |
| Philology, literature, humanities, social sciences and economics, media, art, culture and design | 3.62 (0.82) | 2.6 % (6/227) | 3.1 % (7/227) | 34.8 % (79/227) | 48.9 % (111/227) | 10.6 % (24/227) |

* The mean values between job complexities differed statistically significantly according to the t-test (p-value < 0.0001).

** The mean values between occupational areas did differ statistically significantly according to the t-test (p-value = 0.022)

**Supplementary table 14: Degree of handling multiple simultaneously according to job complexity and occupational area. Mainz and Mainz-Bingen district. 2019–2022.**

| **Variable** | **Mean (SD)**  (1–5) | **Never**  (1) | **Seldom**  (2) | **Sometimes**  (3) | **Often**  (4) | **Always**  (5) |
| --- | --- | --- | --- | --- | --- | --- |
| **Job complexity*** |  |  |  |  |  |  |
| Unskilled activities | 3.01 (1.14) | 13.2 % (10/76) | 17.1 % (13/76) | 32.9 % (25/76) | 28.9 % (22/76) | 7.9 % (6/76) |
| Specialist activities | 3.37 (1.08) | 7.5 % (77/1,024) | 11.8 % (121/1,024) | 29.0 % (297/1,024) | 39.1 % (400/1,024) | 12.6 % (129/1,024) |
| Complex specialist activities | 3.64 (0.95) | 3.0 % (28/930) | 8.5 % (79/930) | 25.6 % (238/930) | 47.2 % (439/930) | 15.7 % (146/930) |
| Highly complex activities | 3.81 (0.92) | 1.5 % (21/1,393) | 7.2 % (100/1,393) | 22.8 % (318/1,393) | 45.7 % (636/1,393) | 22.8 % (318/1,393) |
| **Occupational area**** |  |  |  |  |  |  |
| Agriculture, forestry, farming, and gardening | 3.36 (0.91) | 3.8 % (2/53) | 11.3 % (6/53) | 37.7 % (20/53) | 39.6 % (21/53) | 7.5 % (4/53) |
| Production of raw materials and goods, and manufacturing | 3.59 (0.94) | 2.7 % (10/369) | 10.0 % (37/369) | 26.6 % (98/369) | 46.6 % (172/369) | 14.1 % (52/369) |
| Construction, architecture, surveying, and technical building services | 3.60 (1.00) | 4.8 % (7/145) | 6.9 % (10/145) | 28.3 % (41/145) | 43.4 % (63/145) | 16.6 % (24/145) |
| Natural sciences, geography, and informatics | 3.77 (0.86) | 1.2 % (4/327) | 5.8 % (19/327) | 26.0 % (85/327) | 48.6 % (159/327) | 18.3 % (60/327) |
| Traffic, logistics, safety and security | 3.29 (1.17) | 9.4 % (22/235) | 15.7 % (37/235) | 26.8 % (63/235) | 33.2 % (78/235) | 14.9 % (35/235) |
| Commercial services, trading, sales, the hotel business and tourism | 3.63 (1.00) | 4.9 % (14/288) | 7.3 % (21/288) | 24.7 % (71/288) | 46.5 % (134/288) | 16.7 % (48/288) |
| Business organisation, accounting, law, and administration | 3.64 (0.94) | 2.9 % (29/1,003) | 8.4 % (84/1,003) | 26.4 % (265/1,003) | 46.6 % (467/1,003) | 15.8 % (158/1,003) |
| Health care, the social sector, teaching and education | 3.61 (1.11) | 5.7 % (44/773) | 10.7 % (83/773) | 22.6 % (175/773) | 38.7 % (299/773) | 22.2 % (172/773) |
| Philology, literature, humanities, social sciences and economics, media, art, culture and design | 3.75 (0.92) | 1.8 % (4/227) | 7.0 % (16/227) | 26.0 % (59/227) | 44.9 % (102/227) | 20.3 % (46/227) |

* The mean values between job complexities differed statistically significantly according to the t-test (p-value < 0.0001).

** The mean values between occupational areas did differ statistically significantly according to the t-test (p-value = 0.046)

**Supplementary table 15: Degree of independent problem solving according to job complexity and occupational area. Mainz and Mainz-Bingen district. 2019–2022.**

| **Variable** | **Mean (SD)**  (1–5) | **Never**  (1) | **Seldom**  (2) | **Sometimes**  (3) | **Often**  (4) | **Always**  (5) |
| --- | --- | --- | --- | --- | --- | --- |
| **Job complexity*** |  |  |  |  |  |  |
| Unskilled activities | 3.34 (1.08) | 6.6 % (5/76) | 14.5 % (11/76) | 30.3 % (23/76) | 35.5 % (27/76) | 13.2 % (10/76) |
| Specialist activities | 3.45 (0.98) | 5.8 % (59/1,024) | 7.7 % (79/1,024) | 32.5 % (333/1,024) | 43.5 % (445/1,024) | 10.5 % (108/1,024) |
| Complex specialist activities | 3.72 (0.89) | 2.3 % (21/923) | 6.4 % (59/923) | 24.3 % (224/923) | 51.0 % (471/923) | 16.0 % (148/923) |
| Highly complex activities | 4.05 (0.80) | 1.1 % (15/1,396) | 2.4 % (34/1,396) | 15.7 % (219/1,396) | 52.1 % (728/1,396) | 28.7 % (400/1,396) |
| **Occupational area**** |  |  |  |  |  |  |
| Agriculture, forestry, farming, and gardening | 3.79 (0.76) | 1.9 % (1/53) | 0.0 % (0/53) | 30.2 % (16/53) | 52.8 % (28/53) | 15.1 % (8/53) |
| Production of raw materials and goods, and manufacturing | 3.90 (0.83) | 1.1 % (4/368) | 3.5 % (13/368) | 22.6 % (83/368) | 49.5 % (182/368) | 23.4 % (86/368) |
| Construction, architecture, surveying, and technical building services | 3.86 (0.86) | 2.1 % (3/146) | 3.4 % (5/146) | 21.9 % (32/146) | 51.4 % (75/146) | 21.2 % (31/146) |
| Natural sciences, geography, and informatics | 4.07 (0.75) | 1.2 % (4/328) | 0.6 % (2/328) | 15.5 % (51/328) | 54.9 % (180/328) | 27.7 % (91/328) |
| Traffic, logistics, safety and security | 3.48 (0.99) | 5.1 % (12/235) | 10.6 % (25/235) | 26.8 % (63/235) | 46.4 % (109/235) | 11.1 % (26/235) |
| Commercial services, trading, sales, the hotel business and tourism | 3.67 (1.00) | 4.5 % (13/289) | 7.3 % (21/289) | 22.5 % (65/289) | 47.8 % (138/289) | 18.0 % (52/289) |
| Business organisation, accounting, law, and administration | 3.71 (0.92) | 2.6 % (26/1,001) | 6.5 % (65/1,001) | 25.4 % (254/1,001) | 48.0 % (480/1,001) | 17.6 % (176/1,001) |
| Health care, the social sector, teaching and education | 3.73 (0.98) | 4.4 % (34/770) | 5.2 % (40/770) | 23.0 % (177/770) | 47.4 % (365/770) | 20.0 % (154/770) |
| Philology, literature, humanities, social sciences and economics, media, art, culture and design | 3.78 (0.85) | 1.3 % (3/226) | 5.3 % (12/226) | 25.7 % (58/226) | 49.6 % (112/226) | 18.1 % (41/226) |

* The mean values between job complexities differed statistically significantly according to the t-test (p-value < 0.0001).

** The mean values between occupational areas did differ statistically significantly according to the t-test (p-value = 0.0058).

**Supplementary table 16: Degree of high volumes of information according to job complexity and occupational area. Mainz and Mainz-Bingen district. 2019–2022.**

| **Variable** | **Mean (SD)**  (1–5) | **Never**  (1) | **Seldom**  (2) | **Sometimes**  (3) | **Often**  (4) | **Always**  (5) |
| --- | --- | --- | --- | --- | --- | --- |
| **Job complexity*** |  |  |  |  |  |  |
| Unskilled activities | 2.16 (1.05) | 31.6 % (24/76) | 36.8 % (28/76) | 17.1 % (13/76) | 13.2 % (10/76) | 1.3 % (1/76) |
| Specialist activities | 2.58 (1.10) | 18.4 % (189/1,027) | 31.1 % (319/1,027) | 29.5 % (303/1,027) | 16.5 % (169/1,027) | 4.6 % (47/1,027) |
| Complex specialist activities | 3.01 (1.06) | 7.7 % (71/924) | 24.7 % (228/924) | 34.3 % (317/924) | 25.6 % (237/924) | 7.7 % (71/924) |
| Highly complex activities | 3.32 (1.09) | 5.7 % (79/1,396) | 16.8 % (235/1,396) | 32.4 % (452/1,396) | 30.4 % (424/1,396) | 14.8 % (206/1,396) |
| **Occupational area**** |  |  |  |  |  |  |
| Agriculture, forestry, farming, and gardening | 2.30 (0.98) | 20.8 % (11/53) | 43.4 % (23/53) | 22.6 % (12/53) | 11.3 % (6/53) | 1.9 % (1/53) |
| Production of raw materials and goods, and manufacturing | 2.88 (1.06) | 9.5 % (35/369) | 28.7 % (106/369) | 31.7 % (117/369) | 24.1 % (89/369) | 6.0 % (22/369) |
| Construction, architecture, surveying, and technical building services | 2.90 (1.14) | 11.0 % (16/145) | 27.6 % (40/145) | 31.0 % (45/145) | 20.7 % (30/145) | 9.7 % (14/145) |
| Natural sciences, geography, and informatics | 3.34 (1.05) | 4.6 % (15/327) | 16.5 % (54/327) | 33.0 % (108/327) | 32.4 % (106/327) | 13.5 % (44/327) |
| Traffic, logistics, safety and security | 2.71 (1.12) | 15.3 % (36/235) | 29.8 % (70/235) | 29.4 % (69/235) | 19.6 % (46/235) | 6.0 % (14/235) |
| Commercial services, trading, sales, the hotel business and tourism | 2.73 (1.11) | 15.6 % (45/289) | 25.3 % (73/289) | 36.0 % (104/289) | 16.6 % (48/289) | 6.6 % (19/289) |
| Business organisation, accounting, law, and administration | 3.15 (1.13) | 7.6 % (76/1,003) | 22.5 % (226/1,003) | 28.6 % (287/1,003) | 29.4 % (295/1,003) | 11.9 % (119/1,003) |
| Health care, the social sector, teaching and education | 2.81 (1.15) | 15.3 % (118/772) | 23.8 % (184/772) | 33.5 % (259/772) | 19.2 % (148/772) | 8.2 % (63/772) |
| Philology, literature, humanities, social sciences and economics, media, art, culture and design | 3.33 (1.03) | 4.8 % (11/227) | 14.5 % (33/227) | 36.6 % (83/227) | 31.3 % (71/227) | 12.8 % (29/227) |

* The mean values between job complexities differed statistically significantly according to the t-test (p-value < 0.0001).

** The mean values between occupational areas did not differ statistically significantly according to the t-test (p-value = 0.084)

**Supplementary table 17: Experience of digital and automated work according to job complexity and occupational area. Mainz and Mainz-Bingen district. 2019–2022.**

| **Variable** | **Mean (SD)**  (1–5) | **Negative**  (1) | **Rather negative**  (2) | **Neutral**  (3) | **Rather positive**  (4) | **Positive**  (5) |
| --- | --- | --- | --- | --- | --- | --- |
| **Job complexity*** |  |  |  |  |  |  |
| Unskilled activities | 3.42 (0.72) | 0.0 % (0/72) | 5.6 % (4/72) | 55.6 % (40/72) | 30.6 % (22/72) | 8.3 % (6/72) |
| Specialist activities | 3.58 (0.80) | 1.1 % (11/1,018) | 4.2 % (43/1,018) | 42.7 % (435/1,018) | 39.5 % (402/1,018) | 12.5 % (127/1,018) |
| Complex specialist activities | 3.85 (0.79) | 0.4 % (4/927) | 2.7 % (25/927) | 29.4 % (273/927) | 46.2 % (428/927) | 21.3 % (197/927) |
| Highly complex activities | 4.00 (0.78) | 0.1 % (1/1,395) | 2.8 % (39/1,395) | 21.4 % (299/1,395) | 48.0 % (670/1,395) | 27.7 % (386/1,395) |
| **Occupational area**** |  |  |  |  |  |  |
| Agriculture, forestry, farming, and gardening | 3.25 (0.67) | 0.0 % (0/53) | 11.3 % (6/53) | 54.7 % (29/53) | 32.0 % (17/53) | 1.9 % (1/53) |
| Production of raw materials and goods, and manufacturing | 3.86 (0.80) | 0.3 % (1/367) | 2.7 % (10/367) | 30.5 % (112/367) | 44.1 % (162/367) | 22.3 % (82/367) |
| Construction, architecture, surveying, and technical building services | 3.65 (0.79) | 0.7 % (1/146) | 4.1 % (6/146) | 39.0 % (57/146) | 42.5 % (62/146) | 13.7 % (20/146) |
| Natural sciences, geography, and informatics | 4.26 (0.75) | 0.0 % (0/327) | 1.5 % (5/327) | 13.8 % (45/327) | 41.6 % (136/327) | 43.1 % (141/327) |
| Traffic, logistics, safety and security | 3.60 (0.73) | 0.4 % (1/229) | 3.9 % (9/229) | 40.2 % (92/229) | 46.3 % (106/229) | 9.2 % (21/229) |
| Commercial services, trading, sales, the hotel business and tourism | 3.72 (0.82) | 1.0 % (3/286) | 2.8 % (8/286) | 37.1 % (106/286) | 41.3 % (118/286) | 17.8 % (51/286) |
| Business organisation, accounting, law, and administration | 3.91 (0.77) | 0.5 % (5/1,008) | 1.9 % (19/1,008) | 26.1 % (263/1,008) | 49.0 % (494/1,008) | 22.5 % (227/1,008) |
| Health care, the social sector, teaching and education | 3.64 (0.81) | 0.5 % (4/765) | 5.2 % (40/765) | 38.6 % (295/765) | 41.0 % (314/765) | 14.6 % (112/765) |
| Philology, literature, humanities, social sciences and economics, media, art, culture and design | 3.99 (0.79) | 0.4 % (1/228) | 3.1 % (7/228) | 20.2 % (46/228) | 49.6 % (113/228) | 26.8 % (61/228) |

* The mean values between job complexities differed statistically significantly according to the t-test (p < 0.0001).

** The mean values between occupational areas did not differ statistically significantly according to the t-test (p = 0.09)

**Supplementary table 18: Variable specific Betas (β) and 95 % confidence intervals (95 % CI) for different COPSOQ items. Adjusted for sex, age, date of measurement, and SES. Mainz and Mainz-Bingen district. 2019–2022. (Women)**

|  | **Intention to leave profession/job**  β (95% CI) | **Job satisfaction**  β (95% CI) | **Work engagement**  β (95% CI) | **General health**  β (95% CI) | **Burnout symptoms**  β (95% CI) | **Presenteeism**  β (95% CI) | **Inability to relax**  β (95% CI) |
| --- | --- | --- | --- | --- | --- | --- | --- |
| **Use of digital information** | 1.03 (0.32 to 1.74)  *p =* 0.043  *n =* 2,557 | 0.25 (-0.27 to 0.76)  *p* = 0.35  *n =* 2,507 | -1.42 (-2.03 to -0.81)  *p* < 0.0001  *n =* 2,605 | -0.18 (-0.68 to 0.32)  *p* = 0.47  *n =* 2,892 | 0.98 (0.31 to 1.64)  *p* = 0.0040  *n =* 2,593 | -0.26 (-1.13 to 0.61)  *p* = 0.56  *n =* 2,603 | 0.13 (-0.80 to 1.05)  *p* = 0.79  *n =* 2,599 |
| **Use of digital communication tools** | 1.16 (0.48 to 1.83)  *p =* 0.00078  *n =* 2,552 | 0.54 (0.05 to 1.03)  *p* = 0.031  *n =* 2,502 | -1.13 (-1.72 to -0.55)  *p* = 0.00014  *n =* 2,600 | -0.08 (-0.55 to 0.40)  *p* = 0.76  *n =* 2,587 | 0.73 (0.09 to 1.37)  *p* = 0.024  *n =* 2,589 | -0.56 (-1.39 to 0.27)  *p* = 0.18  *n =* 2,598 | 0.68 (-0.20 to 1.56)  *p* = 0.13  *n =* 2,594 |
| **Digital networking of work processes** | 0.84 (0.24 to 1.45)  *p =* 0.0065  *n =* 2,547 | 0.38 (-0.06 to 0.82)  *p* = 0.094  *n =* 2,498 | -1.07 (-1.59 to -0.55)  *p* < 0.0001  *n =* 2,596 | -0.22 (-0.64 to 0.21)  *p* = 0.31  *n =* 2,582 | 0.54 (-0.03 to 1.11)  *p* = 0.062  *n =* 2,583 | 0.12 (-0.62 to 0.86)  *p* = 0.75  *n =* 2,593 | 0.19 (-0.60 to 0.98)  *p* = 0.63  *n =* 2,589 |
| **Degree of digital work**  (Composite variable) | 1.22 (0.49 to 1.95)  *p* = 0.0010  *n =* 2,544 | 0.47 (-0.06 to 1.00)  *p* = 0.079  *n =* 2,495 | -1.47 (-2.10 to -0.84)  *p* < 0.0001  *n =* 2,593 | -0.23 (-0.74 to 0.29)  *p* = 0.39  *n =* 2,579 | 0.89 (0.21 to 1.58)  *p* = 0.011  *n =* 2,581 | -0.29 (-1.18 to 0.61)  *p* = 0.53  *n =* 2,590 | 0.40 (-0.56 to 1.35)  *p* = 0.42  *n =* 2,586 |
| **Degree of automated work** | 0.77 (-0.30 to 1.84)  *p =* 0.16  *n =* 2,535 | -0.77 (-1.54 to 0.00)  *p* = 0.050  *n =* 2,487 | 0.17 (-0.76 to 1.10)  *p* = 0.73  *n =* 2,583 | -0.27 (-1.03 to 0.49)  *p* = 0.48  *n =* 2,570 | 0.87 (-0.14 to 1.88)  *p* = 0.092  *n =* 2,571 | 0.78 (-0.54 to 2.10)  *p* = 0.25  *n =* 2,581 | 0.60 (-0.80 to 2.00)  *p* = 0.40  *n =* 2,577 |
| **Unforeseeable technical faults** | 3.11 (2.21 to 4.01)  *P* < 0.0001  *n =* 2,529 | -2.43 (-3.07 to -1.78)  *p* < 0.0001  *n =* 2,480 | -2.55 (-3.32 to -1.77)  *p* < 0.0001  *n =* 2,578 | -1.89 (-2.52 to -1.25)  *p* < 0.0001  *n =* 2,564 | 3.67 (2.83 to 4.51)  *p* < 0.0001  *n =* 2,566 | 3.99 (2.89 to 5.09)  *p* < 0.0001  *n =* 2,575 | 3.45 (2.27 to 4.63)  *p* < 0.0001  *n =* 2,571 |
| **Non-influenceable technology** | 1.50 (0.87 to 2.12)  *p* < 0.0001  *n =* 2,454 | -0.88 (-1.33 to -0.43)  *p* = 00014  *n =* 2,413 | -2.06 (-2.60 to -1.53)  *p* < 0.0001  *n =* 2,501 | -0.78 (-1.22 to -0.34)  *p* = 0.00052  *n =* 2,489 | 1.77 (1.18 to 2.36)  *p* < 0.0001  *n =* 2,490 | 0.70 (-0.07 to 1.46)  *p* = 0.074  *n =* 2,498 | 0.46 (-0.36 to 1.28)  *p =* 27  *n =* 2,494 |
| **Demands arising from technical conditions**  (composite variable) | 2.94 (2.06 to 3.81)  *p* < 0.0001  *n =* 2,448 | -2.00 (-2.63 to -1.36)  *p* < 0.0001  *n =* 2,407 | -3.20 (-3.95 to -2.45)  *p* < 0.0001  *n =* 2,495 | -1.65 (-2.28 to -1.04)  *p* < 0.0001  *n =* 2,483 | 3.42 (2.60 to 4.24)  *p* < 0.0001  *n =* 2,484 | 2.48 (1.41 to 3.55)  *p* < 0.0001  *n =* 2,492 | 2.03 (0.88 to 3.18)  *p =* 0.00054  *n =* 2,488 |
| **Innovations requiring knowledge acquisition** | 1.92 (1.10 to 2.75)  *p* < 0.0001  *n =* 2,539 | -0.52 (-1.11 to 0.08)  *p =* 0.087  *n =* 2,491 | -0.37 (-1.09 to 0.34)  *p* = 0.30  *n =* 2,587 | -1.14 (-1.72 to -0.56)  *p* = 0.00012  *n =* 2,573 | 3.34 (2.57 to 4.11)  *p* < 0.0001  *n =* 2,575 | 2.72 (1.71 to 3.73)  *p* < 0.0001  *n =* 2,584 | 3.73 (2.66 to 4.80)  *p* < 0.0001  *n =* 2,580 |
| **Handling multiple tasks simultaneously** | 2.85 (2.05 to 3.66)  *p* < 0.0001  *n =* 2,499 | -2.13 (-2.70 to -1.56)  *p* < 0.0001  *n =* 2,453 | -0.41 (-1.10 to 0.28)  *p* = 0.25  *n =* 2,548 | -1.57 (-2.13 to -1.01)  *p* < 0.0001  *n =* 2,535 | 4.08 (3.34 to 4.82)  *p* < 0.0001  *n =* 2,535 | 4.83 (3.86 to 5.79)  *p* < 0.0001  *n =* 2,544 | 5.29 (4.26 to 6.32)  *p* < 0.0001  *n =* 2,540 |
| **Independent problem solving** | 2.13 (1.24 to 3.01)  *p* < 0.0001  *n =* 2,501 | -1.25 (-1.89 to -0.62)  *p* = 0.00012  *n =* 2,457 | 0.41 (-0.36 to 1.17)  *p* = 0.29  *n =* 2,550 | -1.25 (-1.87 to -0.63)  *p* < 0.0001  *n =* 2,536 | 3.35 (2.53 to 4.17)  *p* < 0.0001  *n =* 2,537 | 3.17 (2.10 to 4.25)  *p* < 0.0001  *n =* 2,546 | 4.03 (2.89 to 5.17)  *p* < 0.0001  *n =* 2,542 |
| **High volumes of information** | 3.08 (2.34 to 3.82)  *p* < 0.0001  *n =* 2,512 | -1.74 (-2.28 to -1.21)  *p* < 0.0001  *n =* 2,466 | -1.25 (-1.89 to -0.61)  *p* = 0.00015  *n =* 2,560 | -1.56 (-2.08 to -1.03)  *p* < 0.0001  *n =* 2,547 | 4.38 (3.70 to 5.06)  *p* < 0.0001  *n =* 2,548 | 3.34 (2.44 to 4.25)  *p* < 0.0001  *n =* 2,557 | 4.68 (3.72 to 5.63)  *p* < 0.0001  *n =* 2,553 |
| **Demands on personal skills**  (composite variable) | 4.57 (3.49 to 5.66)  *p* < 0.0001  *n =* 2,464 | -2.64 (-3.42 to -1.86)  *p* < 0.0001  *n =* 2,423 | -0.82 (-1.76 to 0.12)  *p* = 0.088  *n =* 2,513 | -2.45 (-3.21 to -1.69)  *p* < 0.0001  *n =* 2,501 | 6.87 (5.88 to 7.86)  *p* < 0.0001  *n =* 2,501 | 6.31 (5.00 to 7.62)  *p* < 0.0001  *n =* 2,509 | 8.01 (6.63 to 9.40)  *p* < 0.0001  *n =* 2,505 |
| **Experience of digital and automated work** | -2.48 (-3.53 to -1.42)  *p* < 0.0001  *n =* 2,490 | 3.49 (2.75 to 4.24)  *p* < 0.0001  *n =* 2,449 | 3.53 (2.62 to 4.44)  *p* < 0.0001  *n =* 2,534 | 1.59 (0.85 to 2.36)  *p* < 0.0001  *n =* 2,521 | -2.45 (-3.44 to -1.46)  *p* < 0.0001  *n =* 2,522 | -3.49 (-4.78 to -2.20)  *p* < 0.0001  *n =* 2,531 | -0.76 (-2.14 to 0.62)  *p* = 0.28  *n =* 2,527 |

Adjusted for sex, age, date of measurement, and SES

**Supplementary table 19: Variable specific Betas (β) and 95 % confidence intervals (95 % CI) for different COPSOQ items. Adjusted for sex, age, date of measurement, and SES. Mainz and Mainz-Bingen district. 2019–2022. (Men)**

|  | **Intention to leave profession/job**  β (95% CI) | **Job satisfaction**  β (95% CI) | **Work engagement**  β (95% CI) | **General health**  β (95% CI) | **Burnout symptoms**  β (95% CI) | **Presenteeism**  β (95% CI) | **Inability to relax**  β (95% CI) |
| --- | --- | --- | --- | --- | --- | --- | --- |
| **Use of digital information** | 0.47 (-0.28 to 1.22)  *p =* 0.22  *n =* 2,669 | 1.12 (0.53 to 1.72)  *p* = 0.00023  *n =* 2,591 | -0.37 (-1.10 to 0.37)  *p* = 0.33  *n =* 2,699 | 0.29 (-0.30 to 0.88)  *p* = 0.34  *n =* 2,684 | -0.71 (-1.48 to 0.05)  *p* = 0.069  *n =* 2,686 | -0.52 (-2.52 to -0.53)  *p* = 0.0027  *n =* 2,692 | -0.02 (-1.12 to 1.09)  *p* = 0.98  *n =* 2,692 |
| **Use of digital communication tools** | 0.63 (-0.09 to 1.34)  *p =* 0.086  *n =* 2,666 | 0.83 (0.25 to 1.38)  *p* = 0.0047  *n =* 2,588 | -0.73 (-1.43 to -0.04)  *p* = 0.039  *n =* 2,696 | -0.01 (-0.55 to 0.57)  *p* = 0.98  *n =* 2,681 | -0.13 (-0.86 to 0.60)  *p* = 0.73  *n =* 2,683 | -0.55 (-1.50 to 0.40)  *p* = 0.25  *n =* 2,689 | 1.15 (0.11 to 2.20)  *p* = 0.031  *n =* 2,689 |
| **Digital networking of work processes** | 0.63 (0.01 to 1.24)  *p =* 0.046  *n =* 2,666 | 0.43 (-0.05 to 0.92)  *p* = 0.081  *n =* 2,588 | -0.91 (-1.50 to -0.31)  *p =* 0.0029  *n =* 2,696 | -0.16 (-0.64 to 0.32)  *p* = 0.51  *n =* 2,681 | 0.01 (-0.62 to 0.63)  *p* = 0.99  *n =* 2,683 | -1.14 (-1.95 to -0.33)  *p =* 0.0057  *n =* 2,689 | -0.05 (-0.95 to 0.84)  *p* = 0.91  *n =* 2,689 |
| **Degree of digital work**  (Composite variable) | 0.71 (-0.05 to 1.47)  *p* = 0.067  *n =* 2,665 | 0.91 (0.31 to 1.51)  *p* = 0.0030  *n =* 2,587 | -0.86 (-1.60 to -0.12)  *p* = 0.023  *n =* 2,695 | 0.02 (-0.57 to 0.62)  *p* = 0.94  *n =* 2,680 | -0.29 (-1.06 to 0.49)  *p* = 0.47  *n =* 2,682 | -1.32 (-2.33 to -0.32)  *p* = 0.0098  *n =* 2,688 | 0.40 (-0.72 to 1.51)  *p* = 0.42  *n =* 2,689 |
| **Degree of automated work** | 0.09 (-0.60 to 0.78)  *p =* 0.80  *n =* 2,660 | 0.46 (-1.01 to 0.08)  *p* = 0.097  *n =* 2,583 | 0.06 (-0.61 to 0.74)  *p* = 0.85  *n =* 2,690 | -0.17 (-0.71 to 0.37)  *p* = 0.54  *n =* 2,676 | 0.47 (-0.23 to 1.17)  *p* = 0.19  *n =* 2,677 | 0.72 (-0.19 to 1.63)  *p =* 0.12  *n =* 2,683 | -0.56 (-1.57 to 0.45)  *p* = 0.28  *n =* 2,683 |
| **Unforeseeable technical faults** | 2.46 (1.69 to 3.23)  *p* < 0.0001  *n =* 2,644 | -2.58 (-3.18 to -1.97)  *p* < 0.0001  *n =* 2,567 | -2.78 (-3.53 to -2.03)  *p* < 0.0001  *n =* 2,673 | -1.56 (-2.16 to -0.96)  *p* < 0.0001  *n =* 2,657 | 3.20 (2.42 to 3.98)  *p* < 0.0001  *n =* 2,660 | 2.99 (1.67 to 4.01)  *p* < 0.0001  *n =* 2,666 | 2.02 (0.88 to 3.15)  *p* = 0.00051  *n =* 2,666 |
| **Non-influenceable technology** | 1.74 (1.14 to 2.35)  *p* < 0.0001  *n =* 2,588 | -1.22 (-1.70 to -0.74)  *p* = 0.00014  *n =* 2,517 | -2.10 (-2.69 to -1.51)  *p* < 0.0001  *n =* 2,613 | -1.04 (-1.51 to -0.57)  *p* < 0.0001  *n =* 2,600 | 1.86 (1.34 to 2.57)  *p* < 0.0001  *n =* 2,601 | 0.57 (-0.24 to 1.37)  *p* = 0.17  *n =* 2,608 | 0.62 (-0.27 to 1.51)  *p* = 0.17  *n =* 2,607 |
| **Demands arising from technical conditions**  (composite variable) | 2.84 (2.03 to 3.65)  *p* < 0.0001  *n =* 2,580 | -2.49 (-3.13 to -1.85)  *p* < 0.0001  *n =* 2,509 | -3.33 (-4.12 to -2.55)  *p* < 0.0001  *n =* 2,605 | -1.75 (-2.38 to -1.12)  *p* < 0.0001  *n =* 2,592 | 3.46 (2.64 to 4.28)  *p* < 0.0001  *n =* 2,593 | 2.08 (1.00 to 3.15)  *p* = 0.00016  *n =* 2,600 | 1.62 (0.42 to 2.81)  *p =* 0.0080  *n =* 2,599 |
| **Innovations requiring knowledge acquisition** | 0.36 (-0.43 to 1.16)  *p* = 0.37  *n =* 2,645 | 1.39 (0.77 to 2.02)  *p* < 0.0001  *n =* 2,569 | 1.62 (0.85 to 2.39)  *p* < 0.0001  *n =* 2,674 | 0.53 (-0.09 to 1.15)  *p* = 0.095  *n =* 2,659 | 0.28 (-0.52 to 1.09)  *p =* 0.49  *n =* 2,661 | 0.00 (-1.05 to 1.04)  *p* = 1.00  *n =* 2,667 | 1.61 (0.44 to 2.77)  *p =* 0.0069  *n =* 2,667 |
| **Handling multiple tasks simultaneously** | 1.74 (0.98 to 2.49)  *p* < 0.0001  *n =* 2,610 | -0.70 (-1.31 to -0.09)  *p =* 0.025  *n =* 2,534 | 0.95 (0.20 to 1.69)  *p* = 0.013  *n =* 2,635 | -0.86 (-1.45 to -0.26)  *p =* 0.0051  *n =* 2,620 | 3.19 (2.42 to 3.96)  *p* < 0.0001  *n =* 2,622 | 3.80 (2.80 to 4.80)  *p* < 0.0001  *n =* 2,628 | 5.32 (4.21 to 6.42)  *p* < 0.0001  *n =* 2,628 |
| **Independent problem solving** | 0.72 (-0.16 to 1.60)  *p =* 0.11  *n = 2*,622 | 0.99 (0.29 to 1.69)  *p* = 0.0055  *n =* 2,555 | 2.01 (1.16 to 2.87)  *p* < 0.0001  *n =* 2,650 | 0.02 (-0.67 to 0.71)  *p =* 0.96  *n =* 2,635 | 1.55 (0.66 to 2.45)  *p =* 0.00066  *n =* 2,638 | 2.66 (1.51 to 3.82)  *p* < 0.0001  *n =* 2,644 | 3.27 (1.99 to 4.55)  *p* < 0.0001  *n =* 2,644 |
| **High volumes of information** | 2.31 (1.62 to 3.00)  *p* < 0.0001  *n =* 2,628 | -1.20 (-1.74 to -0.66)  *p =*< 0.0001  *n =* 2,555 | -1.17 (-1.84 to -0.49)  *p* = 0.00069  *n =* 2,656 | -1.58 (-2.11 to -1.04)  *p* < 0.0001  *n =* 2,635 | 3.95 (3.27 to 4.64)  *p* < 0.0001  *n =* 2,643 | 3.51 (2.61 to 4.41)  *p* < 0.0001  *n =* 2,649 | 4.90 (3.91 to 5.90)  *p* < 0.0001  *n =* 2,649 |
| **Demands on personal skills**  (composite variable) | 2.79 (1.70 to 3.89)  *p* < 0.0001  *n =* 2,578 | -0.20 (-1.08 to -0.68)  *p =* 0.65  *n =* 2,505 | 1.32 (0.24 to 2.39)  *p* = 0.017  *n =* 2,603 | -1.17 (-2.04 to -0.30)  *p =* 0.0081  *n =* 2,589 | 4.75 (3.64 to 5.87)  *p* < 0.0001  *n =* 2,591 | 5.27 (3.82 to 6.71)  *p* < 0.0001  *n =* 2,597 | 7.91 (6.32 to 9.50)  *p* < 0.0001  *n =* 2,597 |
| **Experience of digital and automated work** | -1.80 (-2.71 to -0.89)  *p =* 0.00010  *n =* 2,632 | 3.76 (3.05 to 4.48)  *p* < 0.0001  *n =* 2,556 | 3.75 (2.87 to 4.63)  *p* < 0.0001  *n =* 2,660 | 2.08 (1.37 to 2.79)  *p* < 0.0001  *n =* 2,645 | -3.63 (-4.55 to -2.71)  *p* < 0.0001  *n =* 2,647 | -4.67 (-5.87 to -3.47)  *p* < 0.0001  *n =* 2,653 | -1.69 (-3.03 to -0.35)  *p* = 0.014  *n =* 2,653 |

Adjusted for age, date of measurement, and SES
